# Supplementary material for: Modelling the medium-term dynamics of SARS-CoV-2 transmission in England in the Omicron era
Source: Nat Commun. 2022 Aug 19;13:4879. doi: 10.1038/s41467-022-32404-y (PMC9389516; doi:10.1038/s41467-022-32404-y)
Supplement: Supplementary file 1 — Supplementary Information [file 41467_2022_32404_MOESM1_ESM.pdf]

Supplementary Information to: Modelling the medium-term dynamics of SARS-CoV-2 transmission in England in the Omicron era

Rosanna C. Barnard[*1,2], Nicholas G. Davies[1,2], Centre for Mathematical Modelling of Infectious Diseases COVID-19 working group[†1], Mark Jit[1,2] & W. John Edmunds[1,2]

[1] Centre for Mathematical Modelling of Infectious Diseases, London School of Hygiene & Tropical Medicine, Keppel Street, London, WC1E 7HT, UK
[2] Department of Infectious Disease Epidemiology, London School of Hygiene & Tropical Medicine, Keppel Street, London, WC1E 7HT, UK

* Correspondence: rosanna.barnard@lshtm.ac.uk or rosannabarnardresearch@gmail.com
[†] See page 33 of Supplementary Information for working group authors' funding statements

Figure 1 extended caption. Compartmental model diagram. A three-variant deterministic dynamic susceptible-exposed-infected-recovered (SEIR) compartmental model with clinical and sub-clinical

infectious pathways and with vaccination describes SARS-CoV-2 transmission in England. We model seven NHS England regions[64] separately, with each divided into 16 five-year age groups: 0-4 years up to 70-74 years, and 75 years and older. The model incorporates COVID-19 vaccination with two vaccine products (corresponding to the viral-vector (Va) and mRNA-based (Vb) vaccines in use in England), each with first- (Va1, Vb1) and second-dose (Va2, Vb2) protection, and each with lower levels of protection for individuals who received their primary vaccinations but no booster vaccine and have waned (Va2w, Vb2w). All vaccinated individuals have increased protection against different SARS-CoV-2 outcomes compared to susceptible individuals, according to the vaccine product administered and their vaccine dose/waned status (**Supplementary Table 2**). Vaccinated individuals transition from the susceptible (S) compartment into first-dose vaccinated compartments (Va1, Vb1) depending on which vaccine product was received. Following an assumed first-dose duration (**Supplementary Table 5B**), individuals move into second-dose compartments (Va2, Vb2). Following an assumed second-dose duration (**Supplementary Table 5B**), individuals either receive a booster vaccine or transition into waned states (Va2w/Vb2w). We assume that individuals receiving primary courses of a viral-vector vaccine (Va2) or an mRNA vaccine (Vb2) both move to the Vb2 compartment following their booster vaccination, with their second-dose duration beginning again from zero (**Supplementary Table 5B**). This assumptions reflects the fact that all booster vaccinations in England are either the Pfizer/BioNTech or Moderna mRNA vaccines, and evidence finding higher immunogenicity for individuals receiving Pfizer/BioNTech following Oxford-AstraZeneca, compared with individuals receiving both Oxford-AstraZeneca vaccine doses[2]. We model an additional temporary increase in vaccine protection for individuals receiving their first booster vaccine in late 2021/early 2022, which lasts for 180 days. We utilise three separate SARS-CoV-2 variants in the model to capture the introduction and spread of the Alpha, Delta and Omicron variants of concern in England. The model assumes a traditional infection process: upon being infected, individuals leave the susceptible (S), vaccinated (V) or recovered (R) states and move through exposed (E), infectious (I) and recovered (R) states. The latent (L) state is used in addition to the exposed (E) state for breakthrough infections following vaccination and for re- and cross-infections, to achieve additional vaccine protection against disease (**Supplementary Tables 2, 3**). When individuals are infectious (I), they either progress through a subclinical (Is) pathway or a clinical pathway with pre-clinical (Ip) and clinical (Ic) states. Once individuals have been infected and recover (R), we allow for loss of immunity (where individuals return to a susceptible (S) state) and re- and cross-infections (where individuals with immunity become infected, see **Supplementary Table 3**). On the left hand side (yellow shaded background labelled **I**), solid black arrows represent primary vaccinations, solid coloured arrows represent booster vaccinations, and dotted black arrows represent loss of immunity. Coloured dash-dotted arrows denote susceptible and vaccinated individuals becoming infected and moving into the SARS-CoV-2 infection process (grey boxes on red shaded background labelled **II**). Here, solid black arrows denote individuals moving through the infection process and recovering (R) (purple boxes on purple shaded background labelled **III**). Recovered individuals can lose their immunity (dotted black arrows) and return to the susceptible disease state (S) (**Supplementary Table 4**) or be re- or cross-infected (dashed grey arrows) with other SARS-CoV-2 variants (**Supplementary Table 3**).

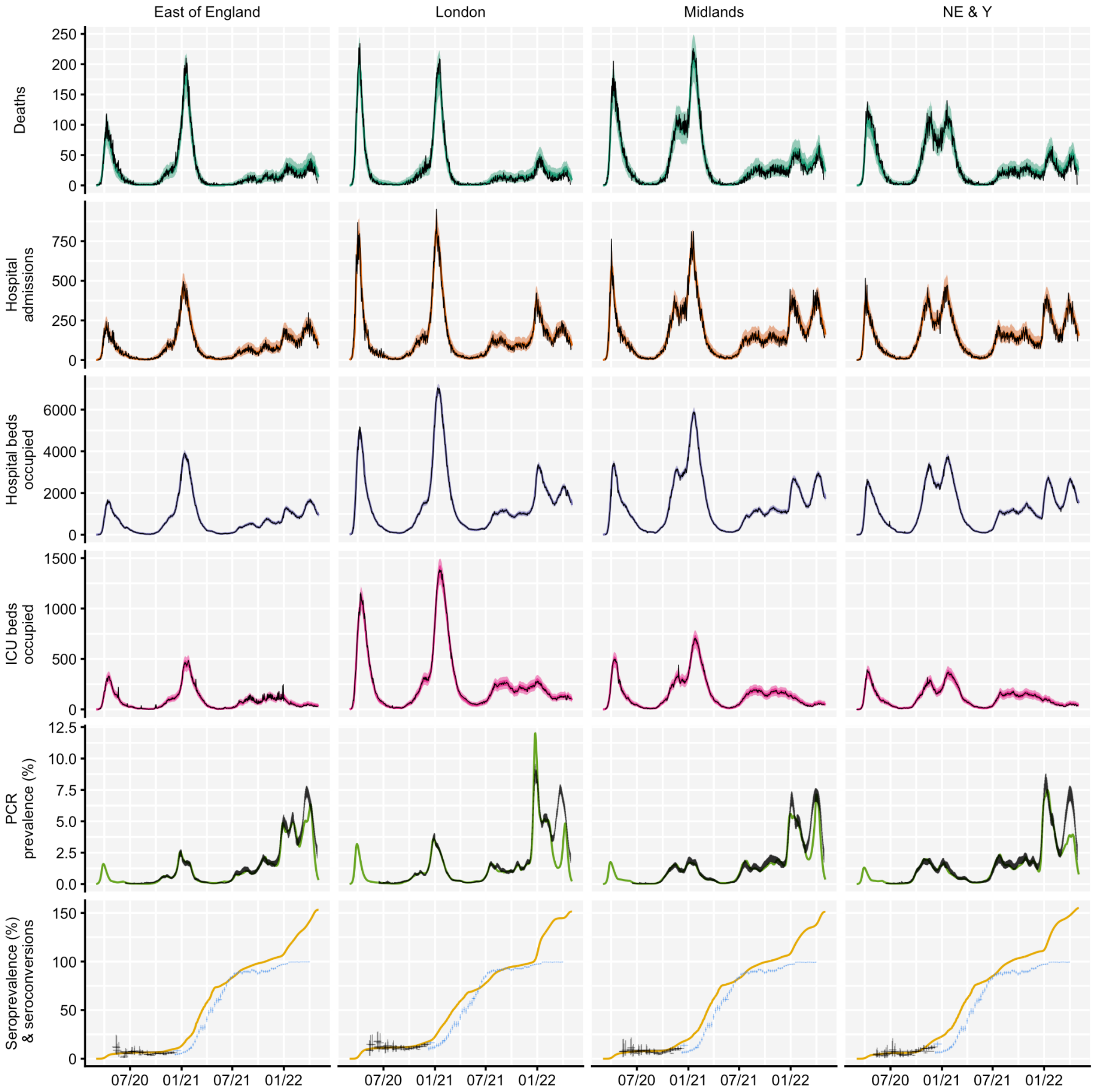

Supplementary Figure 1A. Regional model fit using epidemiological data from NHS England regions between March 2020 and May 2022. Four out of seven NHS England regions are shown here: East of England, London, Midlands and North East & Yorkshire (NE & Y); see **Supplementary Figure 1B** for the remaining three NHS England regions. Black lines show reported data, with vertical black lines showing 95% confidence intervals for PCR prevalence and seroprevalence estimates. Seroprevalence estimates from 1st December 2020 onwards are not used for model fitting and are plotted in blue on top of the modelled cumulative number of seroconversions over time. Coloured lines and shaded areas show medians, 50% and 90% interquantile ranges from the fitted model. COVID-19 deaths data was provided by the UK Health Security Agency (UKHSA) and hospital admissions, hospital and ICU bed occupancy data was provided by NHS England. These data sources are unpublished and not publicly available, but are closely aligned with the UK Government's COVID-19 dashboard[3]. PCR prevalence data was obtained from the Office for National Statistics' COVID-19 Infection Survey (ONS-CIS)[4]. Seroprevalence data was obtained from the UK Biobank[5], REACT-2 study[6] and from the ONS-CIS[4,7]. ICU = intensive care unit. NHS = National Health Service.

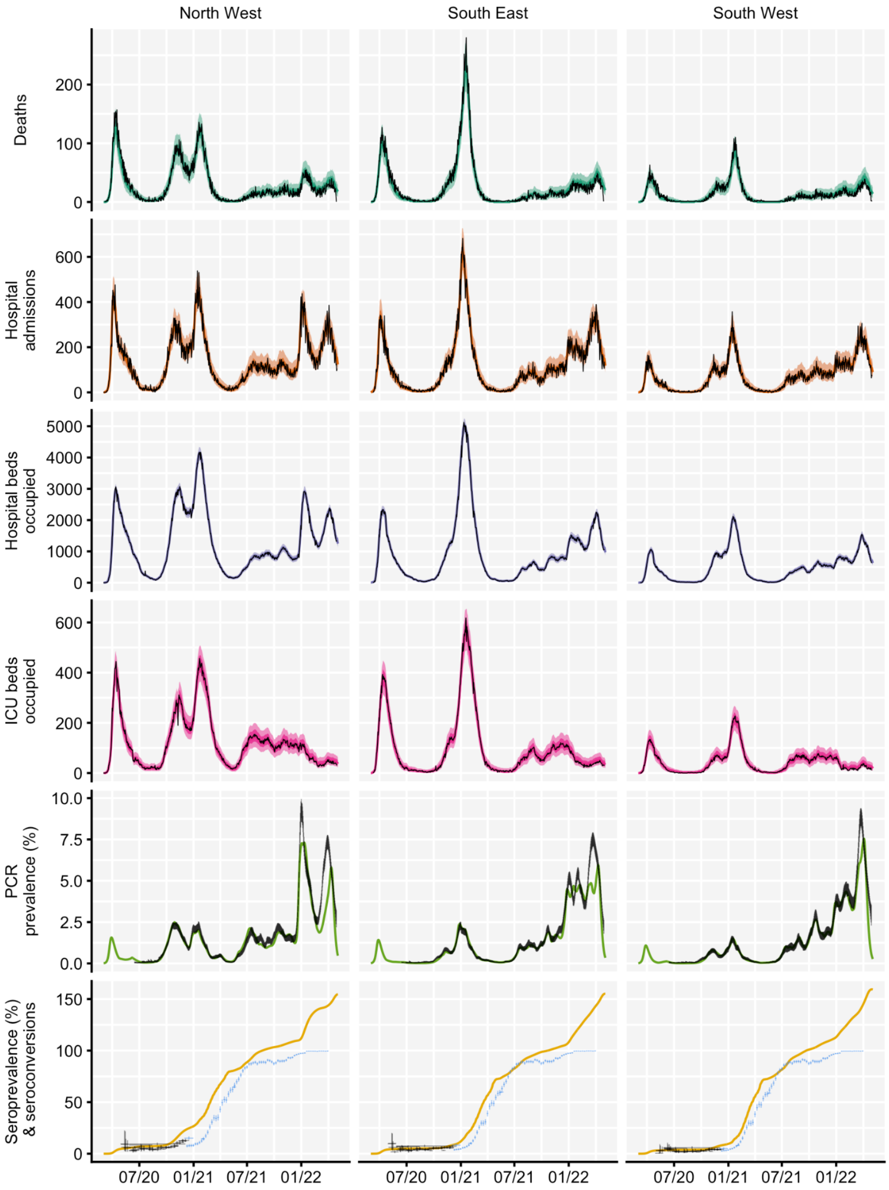

Supplementary Figure 1B. Regional model fit using epidemiological data from NHS England regions between March 2020 and May 2022. Three out of seven NHS England regions are shown here: North West, South East and South West; see **Supplementary Figure 1A** for the remaining four NHS England regions. Black lines show reported data, with vertical black lines showing 95% confidence intervals for PCR prevalence and seroprevalence estimates. Seroprevalence estimates from 1st December 2020 onwards are not used for model fitting and are plotted in blue on top of the modelled cumulative number of seroconversions over time. Coloured lines and shaded areas show medians, 50% and 90% interquantile ranges from the fitted model. COVID-19 deaths data was provided by the UK Health Security Agency (UKHSA) and hospital admissions, hospital and ICU bed occupancy data was provided by NHS England. These data sources are unpublished and not publicly available, but are closely aligned with the UK Government's COVID-19 dashboard[3]. PCR prevalence data was obtained from the Office for National Statistics' COVID-19 Infection Survey (ONS-CIS)[8]. Seroprevalence data was obtained from the UK Biobank[9], REACT-2 study[6] and from the ONS-CIS[4,7,]. ICU = intensive care unit. NHS = National Health Service.

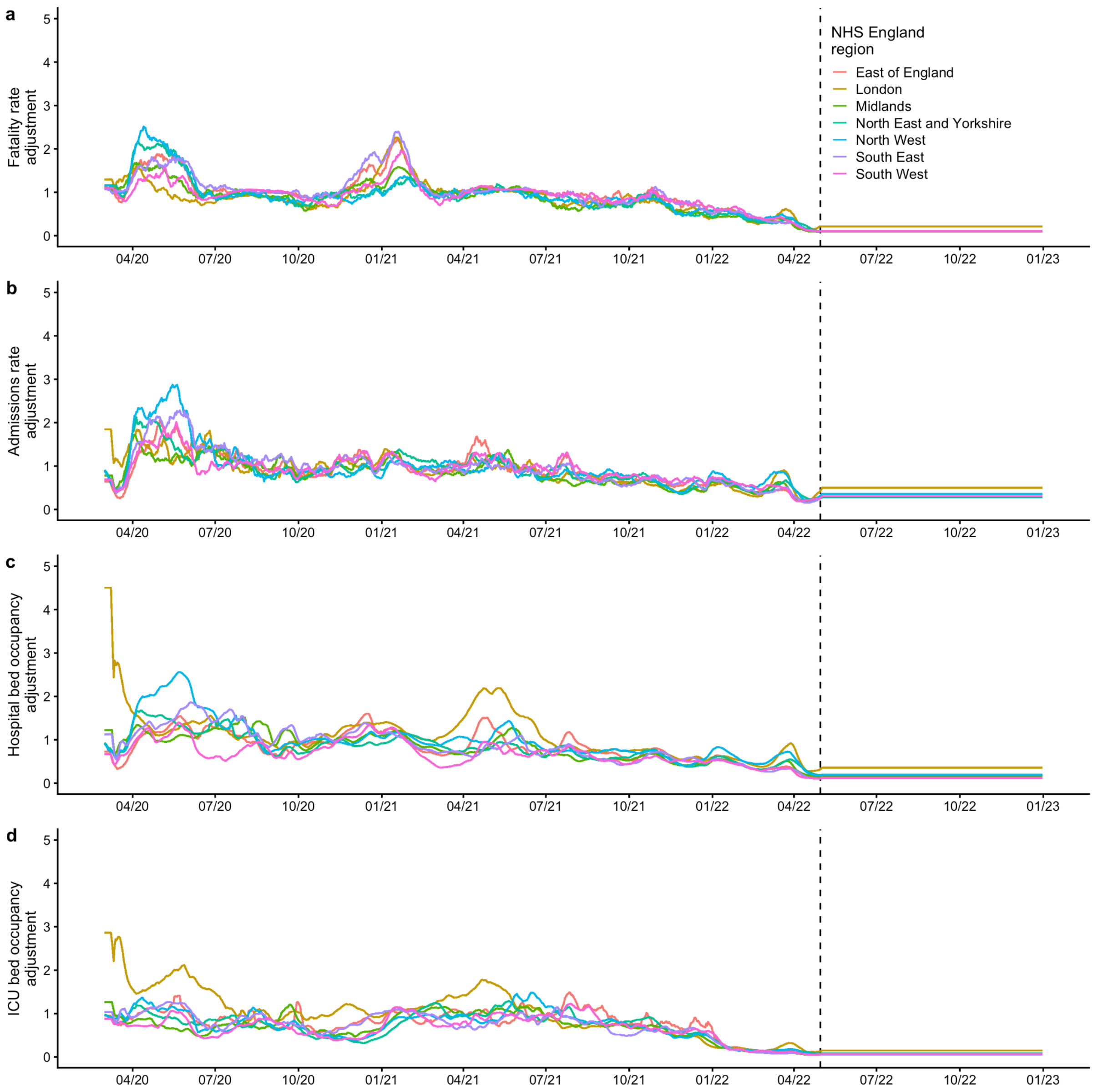

Supplementary Figure 1C. Modelled time-varying adjustments to rates of severe COVID-19 outcomes for the basecase scenario between March 2020 and December 2022. Vertical dashed lines show the final date of data used for model fitting, after which the final measured adjustment value is carried forward for the remainder of each simulation. **(a)** Modelled adjustments to the infection fatality rate over time, plotted for all seven NHS England regions. **(b)** Modelled adjustments to the hospital admission rate over time. **(c)** Modelled adjustments to the hospital bed occupancy rate over time. **(d)** Modelled adjustments to the intensive care unit (ICU) bed occupancy rate over time. NHS = National Health Service.

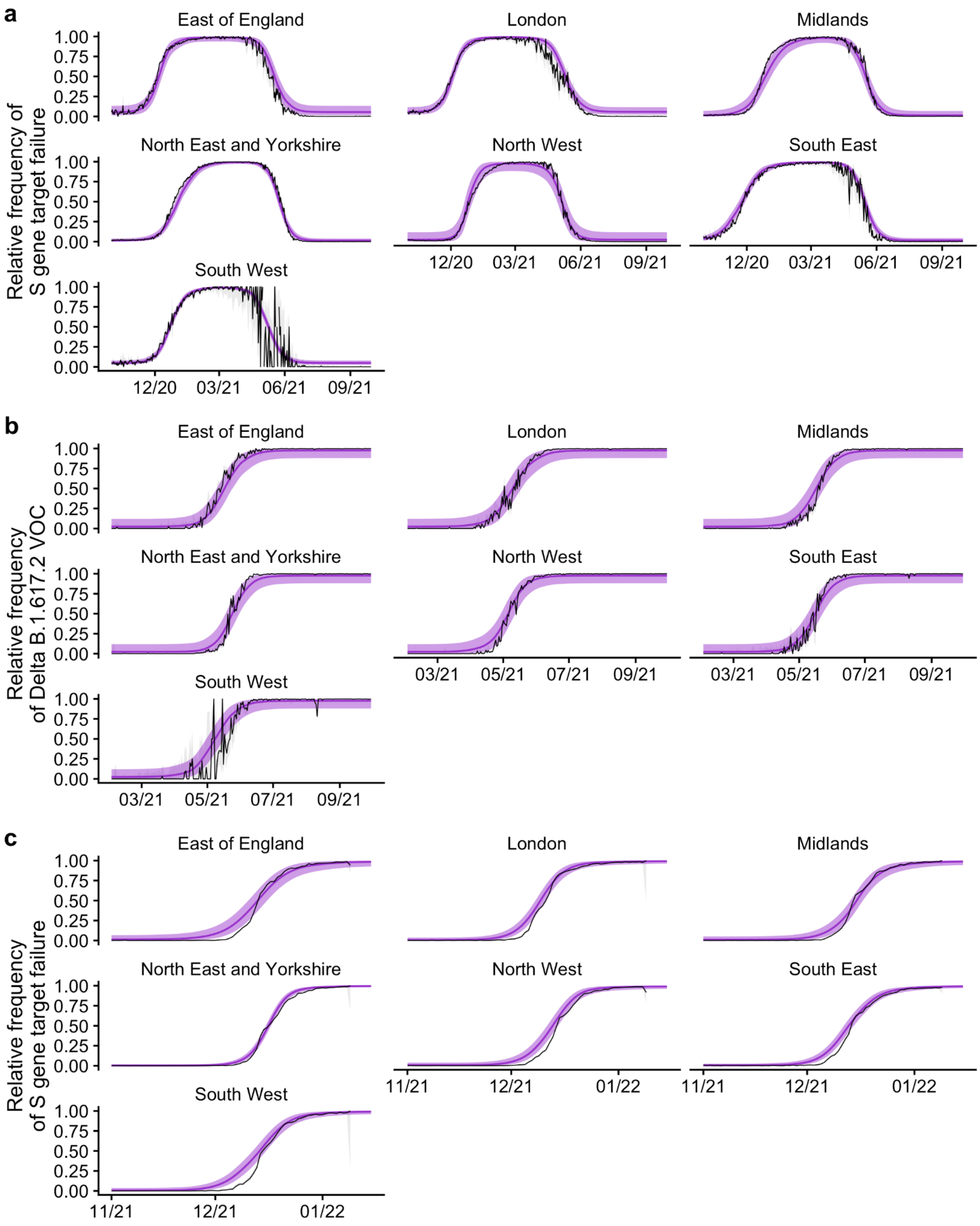

Supplementary Figure 2. Regional model fits to the B.1.1.7 Alpha, B.1.617.2 Delta and B.1.1.529 Omicron variants of concern (VOCs) using S-gene target failure data and genomic sequencing data from NHS England regions between October 2020 and January 2022. (a) For the Alpha B.1.1.7 VOC, we use the frequency of S-gene target failure between October 2020 and September 2021 as a proxy for the proportion of infections attributable to Alpha over time. **(b)** For the Delta B.1.617.2 VOC, we use genomic sequencing data measuring the proportion of sequenced Pillar 2 cases attributable to Delta between February and September 2021. **(c)** For the Omicron B.1.1.529 VOC, we use the frequency of S-gene target failure between November 2021 and January 2022 as a proxy for the proportion of infections attributable to Omicron over time. In all three panels, black lines show reported data, with grey shaded regions showing 95% confidence intervals for the relative frequency of S-gene target failure in Pillar 2 PCR confirmed cases of SARS-CoV-2 (panels **a** and **c**) and the relative frequency of the Delta B.1.617.2 VOC in sequenced Pillar 2 PCR confirmed cases (panel **b**). Coloured lines and shaded areas show medians and 95% interquantile ranges from the fitted model. S-gene target failure and sequencing data was provided by the UK Health Security Agency (UKHSA). These data sources are unpublished and not publicly available. NHS = National Health Service.

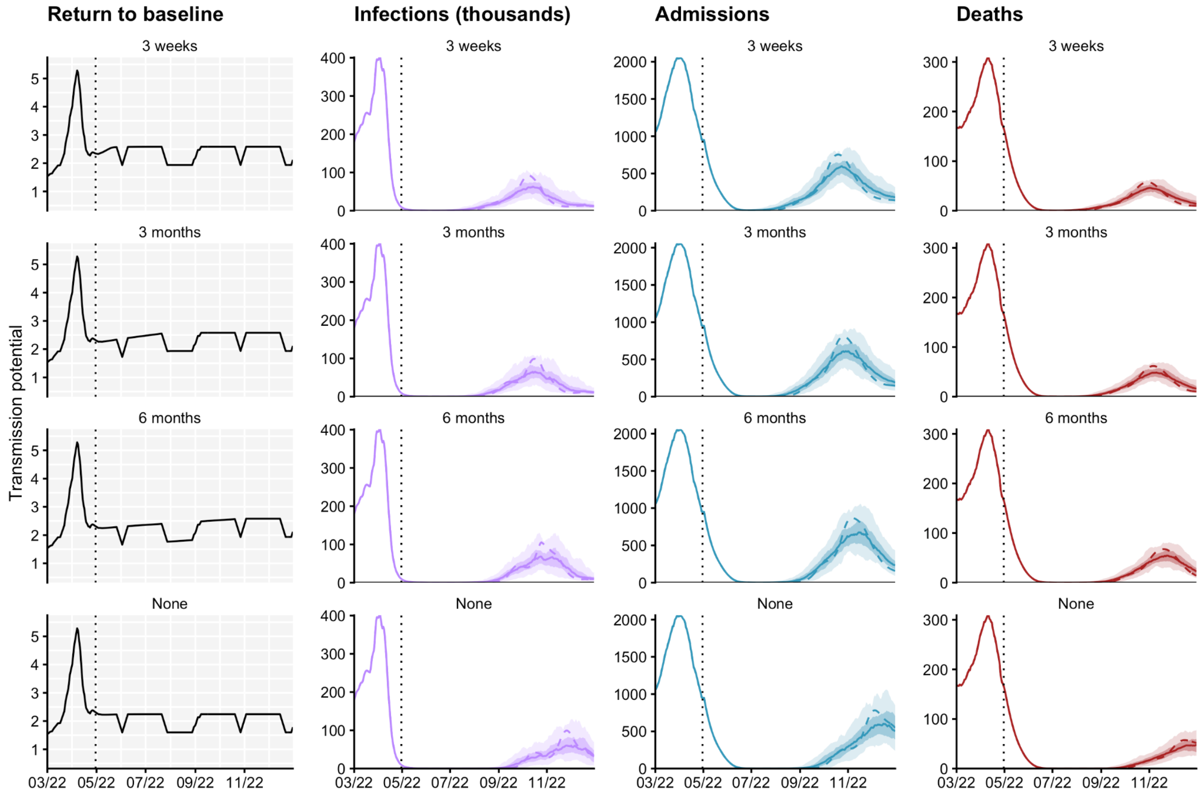

Outcomes from 1st May to 31st December 2022

**Return to baseline**	**Infections**	**Admissions**	**Deaths**
**3 weeks**	*5,120,000 (4,780,000 - 5,400,000)*	*63,500 (59,900 - 67,200)*	*6,030 (5,710 - 6,320)*
**3 months**	*5,180,000 (4,840,000 - 5,430,000)*	*64,000 (60,200 - 67,900)*	*6,070 (5,650 - 6,400)*
**6 months***	*5,290,000 (4,930,000 - 5,590,000)*	*65,200 (60,300 - 69,200)*	*6,130 (5,710 - 6,570)*
**No change**	*4,740,000 (3,970,000 - 5,410,000)*	*52,800 (47,200 - 60,600)*	*4,950 (4,320 - 5,670)*

Supplementary Figure 3. Impact of behaviour change on projected dynamics of SARS-CoV-2 transmission in England from March to December 2022. Top left: Overall transmission potential over time, incorporating mobility data, transmission adjustments and school term dates. Top right: Possible trajectories for COVID-19 infections (thousands), hospital admissions, and deaths are simulated for different rates of return to pre-pandemic baseline levels. The shaded areas and solid lines show the 50% and 90% interquantile ranges and the median for each time point, while the dashed line shows one sample trajectory. The vertical dotted lines denote the end of model fitting and the beginning of model projections. All scenarios assume central waning of vaccine protection (see **Table S4**), measured booster vaccination uptake relative to second dose uptake as of April 2022[10], and 20% seasonality introduced from 1st April 2021. Full details about the assumptions for each scenario are given in **Table 1**. Table: the total number of COVID-19 infections, hospital admissions, and deaths, between 1st May and 31st December 2022, shown to 3 significant figures. The 6-month scenario is marked with an asterisk (*) and corresponds to the basecase scenario.

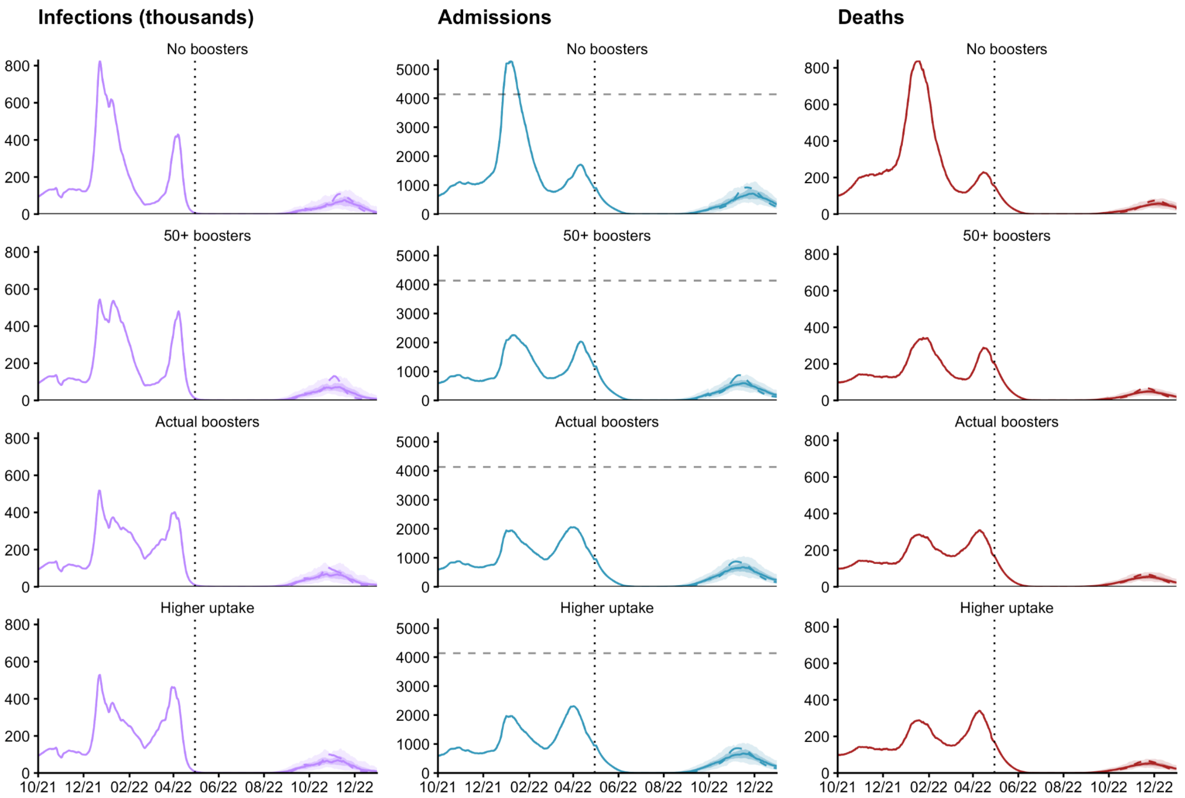

Outcomes from 1st October 2021 to 31st December 2022

Booster scenario	Infections	Admissions	Deaths
**No boosters**	*52,000,000 (51,300,000 - 52,200,000)*	*429,000 (423,000 - 434,000)*	*69,300 (68,800 - 69,800)*
**50+ boosters**	*50,800,000 (50,500,000 - 51,100,000)*	*312,000 (308,000 - 315,000)*	*45,100 (44,800 - 45,400)*
**Actual boosters***	*50,700,000 (50,300,000 - 51,000,000)*	*321,000 (316,000 - 325,000)*	*45,900 (45,400 - 46,300)*
**Higher uptake**	*50,800,000 (50,400,000 - 51,100,000)*	*321,000 (316,000 - 325,000)*	*45,800 (45,400 - 46,200)*

Supplementary Figure 4A. Impact of booster vaccination uptake on projected dynamics of SARS-CoV-2 transmission in England from October 2021 to December 2022. Top: Possible trajectories for COVID-19 infections (thousands), hospital admissions and deaths are simulated until December 2022, with different assumptions used for COVID-19 booster vaccination uptake. From top to bottom, we consider: a counterfactual scenario where booster vaccines were not available, a scenario where booster vaccinations were only offered to individuals aged 50 and above (at 95% uptake), the basecase scenario which is matched to measured uptake of booster vaccines relative to second dose uptake as of April 2022, and an additional counterfactual scenario where higher booster vaccination uptake was achieved (90% for individuals aged 15-49 and 98% for individuals aged 50 and over). Shaded areas and solid lines show the 50% and 90% interquantile ranges and the median for each time point, while the dashed line shows a single sample trajectory. The vertical dotted lines denote the end of model fitting and the beginning of model projections. The horizontal dashed line denotes the maximum number of daily recorded COVID-19 hospitalisations in England[3]. Full details about the assumptions for each scenario are given in **Table 1**. Tables: the total number of COVID-19 infections, hospital admissions, and deaths, between 1st October 2021 and 31st December 2022 and between January and December 2022, shown to 3 significant figures. The actual boosters scenario is marked with an asterisk (*) and corresponds to the basecase scenario.

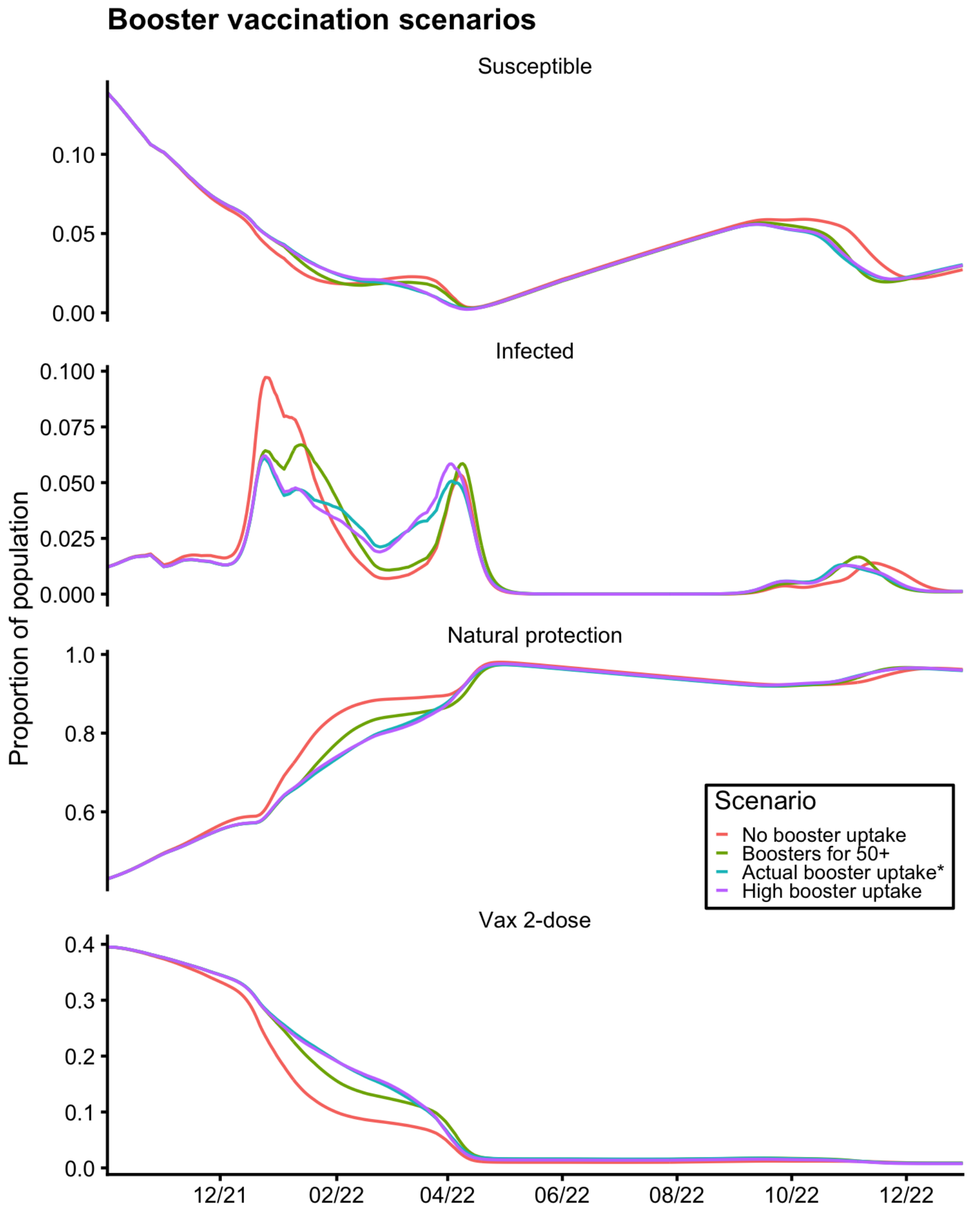

Supplementary Figure 4B. Impact of booster vaccination scenarios on modelled proportions of the population in England in different disease states from October 2021 to December 2022. The modelled proportion of individuals across all ages in England in different disease states (from top to bottom: susceptible, infected, immune (natural protection), and vaccinated with 2-dose levels of protection) between October 2021 and December 2022, shown for one model run from each scenario. Booster vaccination rollout in England started in September 2021, initially targeted to at-risk individuals and individuals aged 50 years and above, 6 months after their previous COVID-19 vaccination. In December 2021 following increasing numbers of Omicron cases, the booster vaccination rollout was accelerated and extended to all individuals aged 18 years and above, with the minimum recommended gap between the previous vaccination and the booster dose shortened to 3 months[11]. Later in December 2022, booster vaccinations were also recommended for individuals aged 16 and 17 years old, at least 3 months following completion of their primary COVID-19 vaccination course[12]. The counterfactual scenario without any booster vaccination uptake (red solid line) is projected to have resulted in a much larger wave of infections (mostly as a result of breakthrough infections in vaccinated individuals) in late 2021 to early 2022.

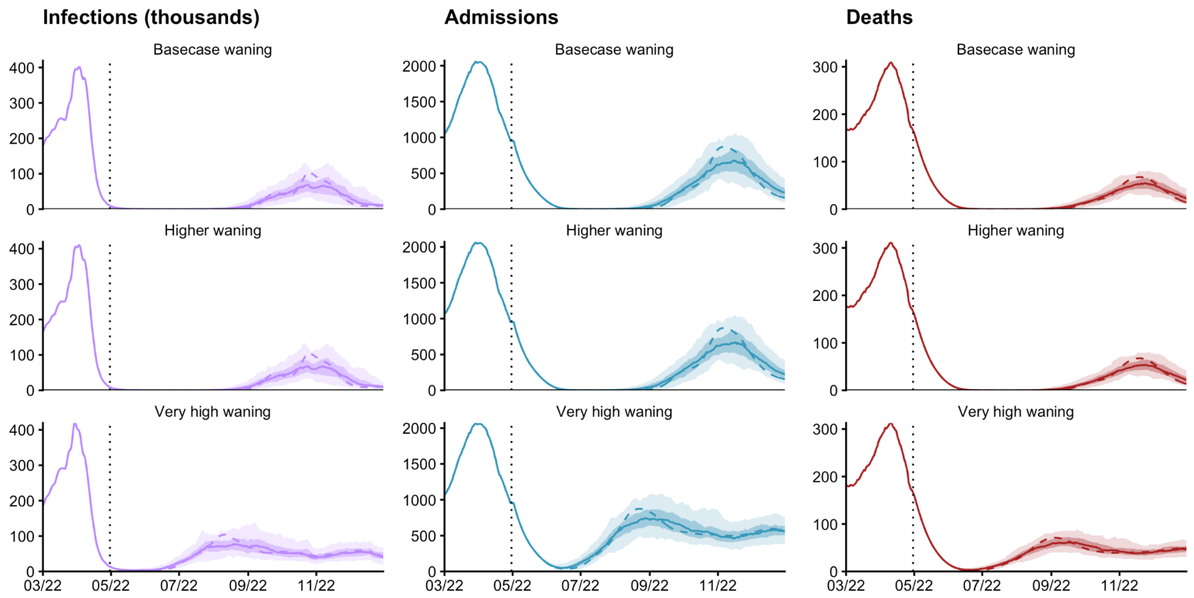

Outcomes from 1st March to 31st December 2022

Waning scenario	Infections	Admissions	Deaths
**Basecase***	*18,900,000 (18,500,000 - 19,200,000)*	*162,000 (157,000 - 166,000)*	*20,100 (19,700 - 20,600)*
**High**	*18,700,000 (18,400,000 - 19,000,000)*	*162,000 (157,000 - 166,000)*	*20,400 (19,900 - 20,800)*
**Very high**	*25,100,000 (24,700,000 - 25,500,000)*	*215,000 (211,000 - 220,000)*	*24,800 (24,400 - 25,200)*

Outcomes from 1st May to 31st December 2022

Waning scenario	Infections	Admissions	Deaths
**Basecase***	*5,290,000 (4,930,000 - 5,590,000)*	*65,200 (60,300 - 69,200)*	*6,130 (5,710 - 6,570)*
**High**	*5,330,000 (5,000,000 - 5,610,000)*	*64,900 (60,500 - 68,900)*	*6,080 (5,660 - 6,500)*
**Very high**	*11,200,000 (10,700,000 - 11,500,000)*	*118,000 (114,000 - 123,000)*	*10,300 (9,940 - 10,700)*

Supplementary Figure 5. Impact of waning immunity on projected dynamics of SARS-CoV-2 transmission in England from March to December 2022. Top: Possible trajectories for COVID-19 infections (thousands), hospital admissions and deaths are simulated until December 2022, with different assumptions used for the rate that immunity (conferred from vaccination and following a prior infection) wanes (see **Table S4**). The shaded areas and solid lines show the 50% and 90% interquantile ranges, and the median for each time point, while the dashed line shows a single sample trajectory. The vertical dotted lines denote the end of model fitting and the beginning of model projections. Full details about the assumptions for each scenario are given in **Table 1**. Tables: the total number of COVID-19 infections, hospital admissions, and deaths, between 1st May and 31st December 2022, and between 1st January and 31st December 2022, shown to 3 significant figures.

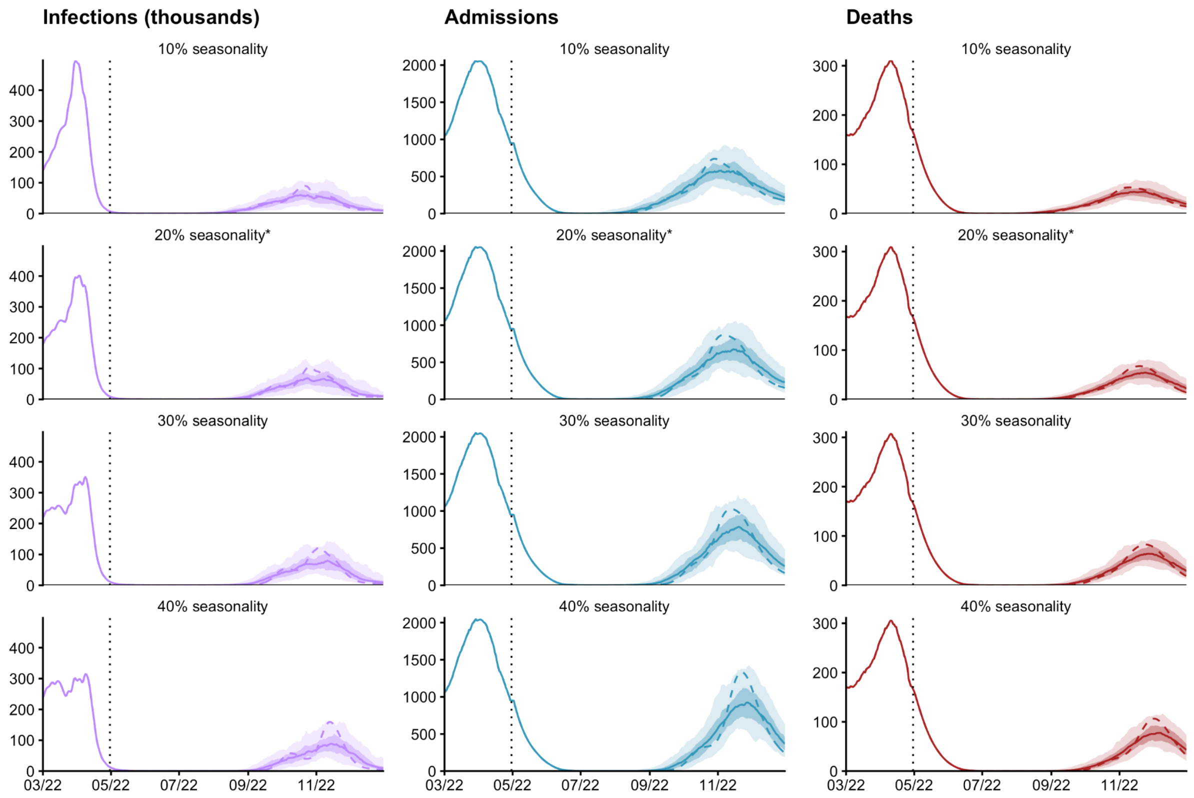

Outcomes from 1st March to 31st December 2022

Seasonality scenario	Infections	Admissions	Deaths
**10%**	*19,300,000 (18,900,000 - 19,600,000)*	*160,000 (155,000 - 164,000)*	*19,700 (19,300 - 20,000)*
**20%***	*18,900,000 (18,500,000 - 19,200,000)*	*162,000 (157,000 - 166,000)*	*20,100 (19,700 - 20,600)*
**30%**	*18,900,000 (18,500,000 - 19,200,000)*	*164,000 (158,000 - 169,000)*	*20,500 (19,900 - 21,000)*
**40%**	*19,400,000 (19,000,000 - 19,700,000)*	*168,000 (161,000 - 174,000)*	*20,900 (20,200 - 21,500)*

Outcomes from 1st May to 31st December 2022

Seasonality scenario	Infections	Admissions	Deaths
**10%**	*5,030,000 (4,610,000 - 5,310,000)*	*63,300 (59,100 - 67,600)*	*5,810 (5,430 - 6,170)*
**20%***	*5,290,000 (4,930,000 - 5,590,000)*	*65,200 (60,300 - 69,200)*	*6,130 (5,710 - 6,570)*
**30%**	*5,580,000 (5,120,000 - 5,880,000)*	*67,800 (61,700 - 72,400)*	*6,510 (5,980 - 7,050)*
**40%**	*5,890,000 (5,460,000 - 6,180,000)*	*72,300 (64,700 - 77,600)*	*6,970 (6,240 - 7,600)*

Supplementary Figure 6. Impact of seasonality on projected dynamics of SARS-CoV-2 transmission in England from March to December 2022. Top: Possible trajectories for COVID-19 infections (thousands), hospital admissions and deaths are simulated until December 2022, with different assumptions made for the extent of seasonality in transmission. From top to bottom: 10%, 20%, 30% and 40% seasonality is introduced from 1st April 2021. The shaded areas and solid lines show the 50% and 90% interquantile ranges, and the median for each time point, while the dashed line shows a single sample trajectory. The vertical dotted lines denote the end of model fitting and the beginning of model projections. Full details about the assumptions for each scenario are given in **Table 1.** Tables: the total number of COVID-19 infections, hospital admissions, and deaths, between 1st March and 31st December 2022 and between 1st May and 31st December 2022, shown to 3 significant figures.

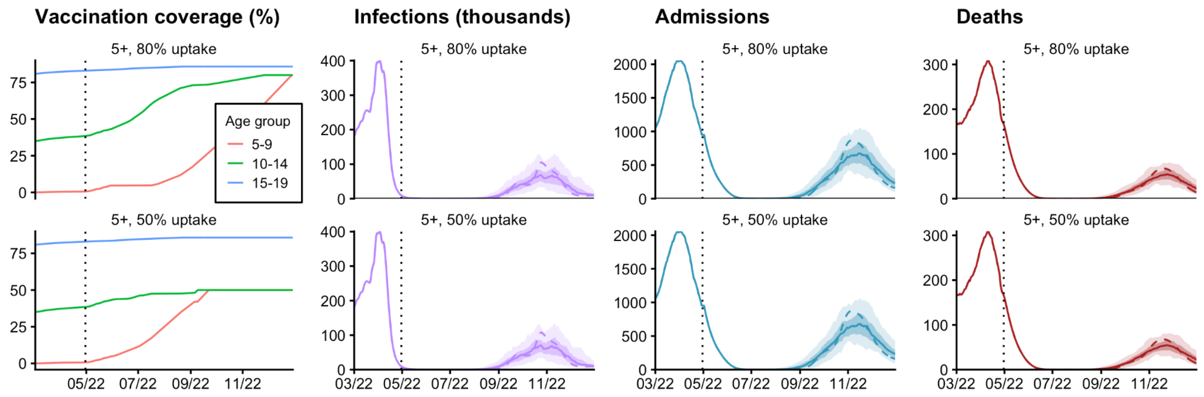

Outcomes from 1st May to 31st December 2022

Vaccination scenario	Infections	Admissions	Deaths
**5+, 80% uptake***	*5,290,000 (4,930,000 - 5,590,000)*	*65,200 (60,300 - 69,200)*	*6,130 (5,710 - 6,570)*
**5+, 50% uptake**	*5,360,000 (5,010,000 - 5,650,000)*	*65,300 (60,600 - 69,300)*	*6,140 (5,710 - 6,580)*

Supplementary Figure 7. Impact of vaccinating adolescents and children on projected dynamics of SARS-CoV-2 transmission in England from March to December 2022. Top left: Vaccination coverage by age group over time for the two scenarios considered, shown for age groups 5-9, 10-14 and 15-19 only. From top to bottom: vaccinating children aged 5 and above at 80% uptake and at 50% uptake. Top right: Possible trajectories for COVID-19 infections (thousands), hospital admissions and deaths are simulated until December 2022, with different assumptions made for the levels of vaccination coverage in children aged 5 years and above. The shaded areas and solid lines show the 50% and 90% interquantile ranges, and the median for each time point, while the dashed line shows a single sample trajectory. The vertical dotted lines denote the end of model fitting and the beginning of model projections. Full details about the assumptions for each scenario are given in **Table 1**. Tables: the total number of COVID-19 infections, hospital admissions, and deaths, between 1st May and 31st December 2022, shown to 3 significant figures.

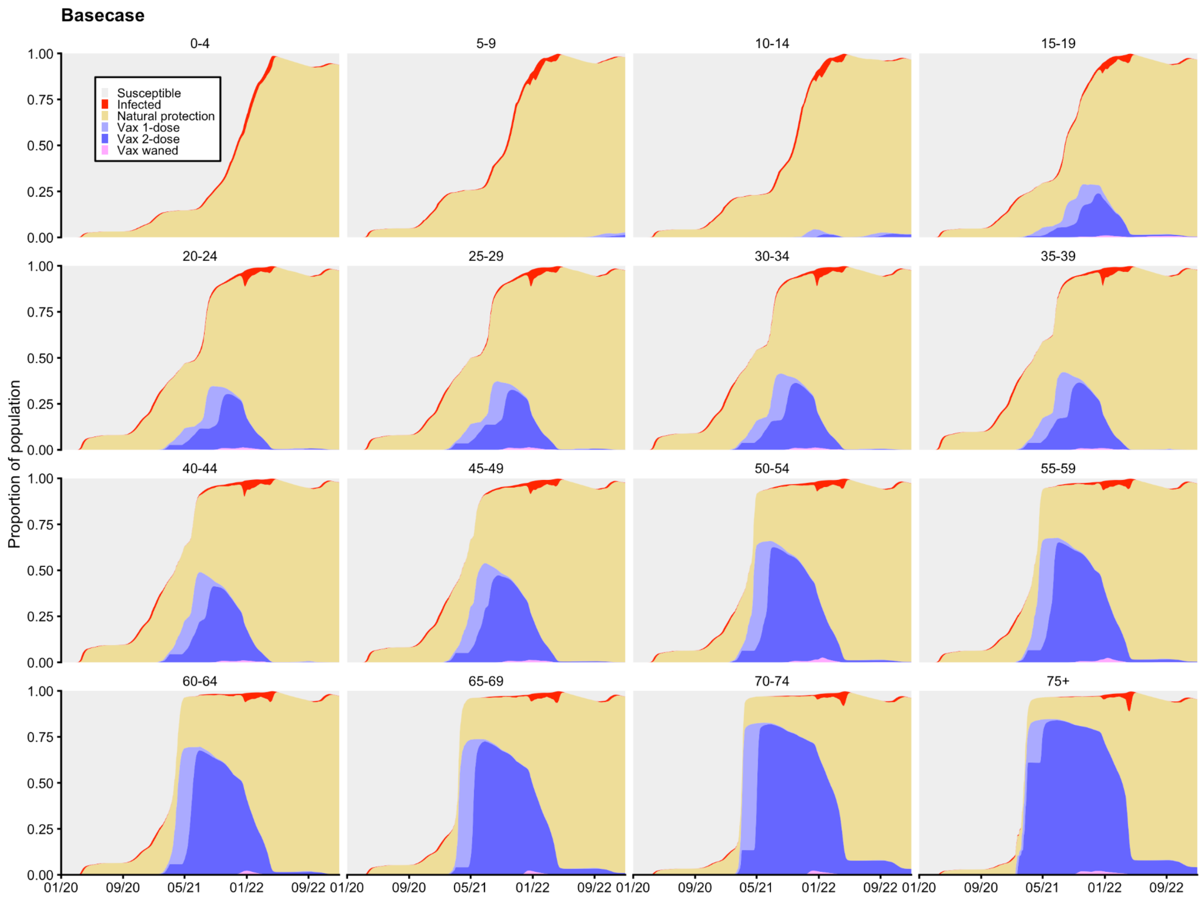

Supplementary Figure 8. Modelled distribution of disease states over time for each 5-year age group, amalgamated across England from January 2020 to December 2022. The basecase scenario is shown here, which includes the model fitting period (up to 13th May 2022) and the basecase model projection until December 2022. Each panel shows the proportion of individuals in each age group who are: currently susceptible (grey), currently infectious (red), naturally protected (including individuals who have been vaccinated and have natural protection due to infection prior to or after their vaccination) (yellow), vaccine protected with 1 dose (light purple), vaccine protected with 2 doses (including individuals who have received booster doses) (dark purple), and partially vaccine protected with waned vaccine protection approximately six months after the second dose (and having not received any booster vaccination) (pink). Note that, due to waning of both natural and vaccine protection back to the fully-susceptible state, the susceptible proportion does not represent the fraction of each age group that has never been infected or vaccinated, as it includes some people who have been previously infected and/or vaccinated but have completely lost their protection.

Supplementary Table 1A - Fitted model estimates for the relative transmissibility of the Alpha B.1.1.7 variant (compared to pre-existing SARS-CoV-2 variants), Delta B.1.617.2 variant (compared to Alpha), and the Omicron B.1.1.529 variant's BA.1 sublineage (compared to Delta).

NHS England region	Alpha B.1.1.7 transmissibility (relative to pre-existing variants)	Delta B.1.617.2 transmissibility (relative to Alpha B.1.1.7)	Omicron B.1.1.529 sublineage BA.1 transmissibility (relative to Delta B.1.617.2)
East of England	1.862965	1.627675	1.1351497
London	1.835242	1.583826	1.5852839
Midlands	1.513771	1.588105	1.1198891
North East & Yorkshire	1.591191	1.740032	1.3439809
North West	2.139368	1.657270	0.9028017
South East	1.466895	1.657890	1.5161782
South West	1.694188	1.527031	1.2348388

Supplementary Table 1B - Fitted model estimates for the relative transmissibility of the Delta B.1.617.2 variant and the Omicron B.1.1.529 BA.1 sublineage compared to pre-existing SARS-CoV-2 variants (i.e. wild type and D614G). The third column shows the overall relative transmissibility of the Omicron BA.2 sublineage, given the fitted values for previously circulating variants and Omicron sublineage BA.1, and our assumption that BA.2 is 50% more transmissible than BA.1.

NHS England region	Delta relative to wild type / D614G variants	Omicron BA.1 relative to wild type / D614G variants	Omicron BA.2 relative to wild type / D614G variants
East of England	3.032302	3.442117	5.163176
London	2.906704	4.607951	6.911926
Midlands	2.404027	2.692244	4.038366
North East & Yorkshire	2.768723	3.721111	5.581666
North West	3.545509	3.200892	4.801338
South East	2.431951	3.687271	5.530907
South West	2.587078	3.194625	4.791937

Supplementary Table 2 - Assumptions for overall vaccine effectiveness against all SARS-CoV-2 outcomes, percentages rounded to 2 significant figures.

**Outcome**	**Variant name**	**Vaccine effectiveness**							
		*Pfizer-BioNTech**				*Oxford-AstraZeneca*			
		1 dose	2 doses	2 doses + boost	2 doses + waned (i.e. no booster)	1 dose	2 doses	2 doses + boost	2 doses + waned (i.e. no booster)
Infection	pre-Alpha & Alpha	70%	85%	N/A[±]	48%	70%	75%	N/A[±]	43%
	Delta[^]	62%	80%	91%	45%	43%	63%	91%	36%
	Omicron[†]	34%	44%	66%	25%	24%	35%	66%	20%
Disease	pre-Alpha & Alpha	70%	90%	N/A[±]	68%	70%	80%	N/A[±]	60%
	Delta[^]	62%	81%	92%	61%	52%	65%	92%	49%
	Omicron[†]	34%	47%	68%	47%	36%	38%	68%	36%
Hospital admission	pre-Alpha & Alpha	85%	95%	N/A[±]	86%	85%	90%	N/A[±]	83%
	Delta[^]	92%	96%	99%	84%	84%	93%	99%	78%
	Omicron[†]	77%	84%	93%	68%	66%	77%	93%	61%
Mortality	pre-Alpha & Alpha	85%	95%	N/A[±]	86%	85%	95%	N/A[±]	83%
	Delta[^]	92%	96%	99%	84%	95%	95%	99%	78%
	Omicron[†]	77%	84%	93%	68%	66%	77%	93%	61%
Onward transmission	pre-Alpha & Alpha	47%	47%	N/A[±]	30%	47%	47%	N/A[±]	29%
	Delta[^]	24%	37%	37%	24%	5%	27%	37%	17%
	Omicron[†]	24%	37%	37%	24%	5%	27%	37%	17%
Delay to efficacy		28 days	14 days	Immediate	Immediate	28 days	14 days	Immediate	Immediate

*We assume that the Moderna mRNA-1273 vaccine confers the same levels of protection as the Pfizer-BioNTech vaccine.

[^]For first- and second-dose vaccine effectiveness with the Delta B.1.617.2 variant, we either scale the equivalent vaccine effectiveness assumption for pre-Alpha and Alpha variants by the unweighted mean change in protection from Alpha to Delta as measured by the references in **Supplementary Table 7**, or assume equivalent values from the previous equivalent dose or the previous level of protection.

[†]For first- and second-dose vaccine effectiveness against infection with the Omicron variant, we assume a 5.5-fold reduction in neutralisation between the Delta and the Omicron variant (the escape low scenario from our previous work on Omicron[13]), which results in an approximate 45% reduction in vaccine protection against infection. We use these values against infection and scale them up to protection against disease, hospitalisation and mortality. For disease, we use the same conditional protection against disease given infection as for the Delta B.1.617.2 variant (back-calculated overall protection against disease is quoted here). For protection against hospitalisation and mortality, we use Khoury et al.'s[14] modelled relationship between efficacy against any infection and efficacy against severe infection to scale up our assumptions for protection against infection with Omicron to higher levels of protection against these severe modelled outcomes. For protection against onward transmission, we use the same assumptions as for the Delta B.1.617.2 variant.

[±]We don't list vaccine effectiveness assumptions for boosted individuals against pre-Alpha B.1.1.7 and Alpha B.1.1.7, as booster vaccinations were offered from September 2021 onwards, once the Delta B.1.617.2 variant had become the dominant variant in England.

Supplementary Table 3 - Assumptions for cross protection against SARS-CoV-2 infection and sequential disease outcomes given immunity from a prior infection with SARS-CoV-2

**Outcome**	**Variant name**	**Cross protection given immunity from infection with wildtype / pre-Alpha**	**Cross protection given immunity from infection with Alpha**	**Cross protection given immunity from infection with Delta**	**Cross protection given immunity from infection with Omicron**
Infection	pre-Alpha & Alpha	100%	100%	100%	100%
	Delta	100%	100%	100%	100%
	Omicron†	55%	55%	55%	100%
Disease	pre-Alpha & Alpha	100%	100%	100%	100%
	Delta	100%	100%	100%	100%
	Omicron†	57%	57%	57%	100%
Hospitalisation	pre-Alpha & Alpha	100%	100%	100%	100%
	Delta	100%	100%	100%	100%
	Omicron†	89%	89%	89%	100%
Mortality	pre-Alpha & Alpha	100%	100%	100%	100%
	Delta	100%	100%	100%	100%
	Omicron†	89%	89%	89%	100%
Onward transmission	pre-Alpha & Alpha	47% (100%)+	47% (100%)+	47% (100%)+	47% (100%)+
	Delta	37% (100%)+	37% (100%)+	37% (100%)+	37% (100%)+
	Omicron†	37%	37%	37%	47% (100%)+

+Where protection against infection is 100%, effective protection against sequential SARS-CoV-2 outcomes (disease, hospital admission, mortality and onward transmission) is also 100%, even if model parameters are set to lower levels of protection.

†For cross protection against infection with the Omicron B.1.1.529 variant, we assume a 5.5-fold reduction in neutralisation between the Delta and the Omicron variant (the escape low scenario from our previous work[13]), which results in an approximate 45% reduction in cross protection against infection with Delta B.1.617.2. We use these values against infection and scale them up to protection against disease, hospitalisation and mortality. For disease, we use the same conditional protection against disease given infection as for the Delta B.1.617.2 variant for individuals who were vaccinated with two doses of Pfizer (see **Supplementary Table 2**) (back-calculated overall protection against disease is quoted here). For cross protection against hospitalisation and mortality, we use Khoury et al.'s[14] modelled relationship between efficacy against any infection and efficacy against severe infection to scale up our assumptions for cross protection against infection with Omicron to higher levels of protection against these severe modelled outcomes. For protection against onward transmission, we use the same assumptions as for the Delta B.1.617.2 variant for individuals who were vaccinated with two doses of Pfizer (see **Supplementary Table 2**).

Supplementary Table 4 - Waning immunity scenarios. Modelling assumptions for the rates of waning immunity. All rates shown here correspond to the rates at which individuals with some form of immunity (either from vaccination or from a prior infection) lose their immunity and return to a fully susceptible disease state. Default waning values are used for the majority of scenarios, including the basecase (see main manuscript **Table 1**). The high waning scenario assumes a non-zero rate of waning for individuals with second-dose / second-dose + boosted levels of protection (wva2, wvb2), whereas the central scenario assumes no waning for these categories of individuals. The very high waning scenario assumes the same loss of protection as the high waning scenario but in half the amount of time.

**Parameter name(s)**	**Description**	**Default values (central waning)**	**High waning**	**Very high waning**
`wn, wn2, wn3`	Rate of waning out of recovered compartment and back to susceptible, for all modelled SARS-CoV-2 strains	log(0.85)/-365, corresponding to exponential waning with a 15% loss of protection after 1 year^	log(0.85)/-365, corresponding to exponential waning with a 15% loss of protection after 1 year	log(0.85)/-182.5, corresponding to exponential waning with a 15% loss of protection after 6 months
`wva1`	Rate of waning out of first-dose AstraZeneca vaccinated compartment	0	0	0
`wva2`	Rate of waning out of second-dose (and second-dose + boosted) AstraZeneca vaccinated compartment	0	log(0.851)/-140	log(0.851)/-70
`wva3`	Rate of waning out of second-dose + waned AstraZeneca vaccinated compartment	log(0.851)/-140*	log(0.851)/-140	log(0.851)/-70
`wvb1`	Rate of waning out of first-dose mRNA vaccinated compartment	0	0	0
`wvb2`	Rate of waning out of second-dose (and second-dose + boosted) mRNA vaccinated compartment	0	log(0.923)/-140	log(0.923)/-70
`wvb3`	Rate of waning out of second-dose + waned mRNA vaccinated compartment	log(0.923)/-140*	log(0.923)/-140	log(0.923)/-70

^We referred to a number of studies looking at the level of protection against reinfection with SARS-CoV-2[15]. An unweighted mean across these studies finds approximately 85.74% protection against reinfection with SARS-CoV-2 after 27.76 weeks. When individuals in the model wane, they lose all remaining protection against SARS-CoV-2 outcomes of all types. Our central assumptions therefore assume that the rate at which individuals lose all their protection is slower than that at which reinfections might occur.
*We used vaccine-specific measured reductions in protection against hospitalisation over 20 weeks from Andrews et al.[16]

Supplementary Table 5A - Details of fitted model parameters. The initial DE-MCMC fitting was done independently for each NHS England region, with 12500 burn-in iterations and 1250 final iterations (13750 iterations total for each region).

Parameter	Description	Prior distribution or assumed value / distribution	Notes
`tS`	Start date of wild-type SARS-CoV-2 epidemic in days after 1 January 2020	$\sim uniform(0, 60)$ (i.e. 1st January - 1st March 2020)	Determines the date at which seeding begins in a region; starting on this date, one random individual per day contracts SARS-CoV-2, repeated for 28 days
`v2_when`	Start date of Alpha B.1.1.7 SARS-CoV-2 variant epidemic in days after 1 January 2020	$\sim uniform(144, 365)$ (i.e. 24th May - 31st December 2020)	Determines the date at which the novel SARS-CoV-2 variant is introduced into a region; on this date, ten random individuals contract the Alpha B.1.1.7 SARS-CoV-2 variant
`v3_when`	Start date of Delta B.1.617.2 SARS-CoV-2 variant epidemic in days after 1 January 2020	$\sim uniform(366, 486)$ (i.e. 1st January - 1st May 2021)	Determines the date at which the novel SARS-CoV-2 variant is introduced into a region; on this date, ten random individuals contract the Delta B.1.617.2 SARS-CoV-2 variant
`v4_when`	Start date of Omicron B.1.1.529 SARS-CoV-2 variant epidemic in days after 1 January 2020	$\sim normal(685, 7)$, ≥ 670 and ≤ 700 (i.e. 1st November 2021 - 1st December 2021)	Determines the date at which the novel SARS-CoV-2 variant is introduced into a region; on this date, ten random individuals contract the Omicron B.1.1.529 SARS-CoV-2 variant, repeated for 14 days
`u`	Basic susceptibility to infection	$\sim normal(0.09, 0.02)$, ≥ 0.04 and ≤ 0.2	Determines basic reproduction number R_0.
`v2_relu`	Relative transmissibility of Alpha B.1.1.7 variant, compared to pre-existing variants	$\sim lognormal(0, 0.4)$, ≥ 0.25 and ≤ 4	Determines the transmission advantage that the second SARS-CoV-2 variant in the model, parameterised for the Alpha B.1.1.7 variant, has over pre-existing variants.
`v3_relu`	Relative transmissibility of Delta B.1.617.2 variant, compared to Alpha B.1.1.7	$\sim lognormal(0, 0.4)$, ≥ 0.25 and ≤ 4	Determines the transmission advantage that the third SARS-CoV-2 variant in the model, parameterised for the Delta B.1.617.2 variant, has over the Alpha B.1.1.7 variant.
`v4_relu`	Relative transmissibility of Omicron B.1.1.529 variant, compared to Delta B.1.617.2	$\sim lognormal(0.4, 0.1)$, ≥ 0.25 and ≤ 4	Determines the transmission advantage that the fourth SARS-CoV-2 variant in the model, parameterised for the Omicron B.1.1.529 variant, has over the Delta B.1.617.2 variant. N.B. although the compartmental model accounts for three SARS-CoV-2 variants in total (see main manuscript **Figure 1)**; we repurpose the first modelled variant (which originally described wildtype and other SARS-CoV-2 variants circulating prior to the Alpha B.1.1.7 variant) for the new Omicron variant on 22nd September 2021.
`v2_hosp_rlo`<br>`v2_icu_rlo`<br>`v2_cfr_rlo`	Relative log-odds of hospitalisation, ICU admission and death for the Alpha B.1.1.7 variant, compared to pre-existing variants	$\sim normal(0, 0.1)$, $\geq$ -4 and ≤ 4	Vague priors

`v2_sgtf0`	The proportion of wild-type SARS-CoV-2 that produce S gene target failure (i.e. the “false positive” rate of identifying Alpha B.1.1.7 by SGTF frequency)	$\sim beta(1.5, 15)$	Vague prior
`v2_disp`	Controls variance in the distribution for fitting model-predicted Alpha B.1.1.7 frequency to SGTF data	$\sim exponential(10, 10)$, ≥ 0 and ≤ 0.25	Vague prior
`v4_sgtf0`	The proportion of non-Omicron SARS-CoV-2 that produce S gene target failure (i.e. the “false positive” rate of identifying Omicron B.1.1.529 by SGTF frequency)	$\sim beta(1.5, 15)$	Vague prior
`v4_disp`	Controls variance in the distribution for fitting model-predicted Omicron B.1.1.529 frequency to SGTF data	$\sim exponential(10, 10)$, ≥ 0 and ≤ 0.25	Vague prior
`death_mean`	Mean delay in days from start of infectious period to death	$\sim normal(15, 2)$, ≥ 5 and ≤ 30	The delay itself is assumed to follow a gamma distribution with shape parameter 2.2, and mean `death_mean`. Prior and shape of distribution informed by analysis of CO-CIN data[17].
`hosp_admission`	Mean delay in days from start of infectious period to hospital admission	$\sim normal(8, 1)$, ≥ 4 and ≤ 20	Delay is assumed to follow a gamma distribution with shape parameter 0.71 and mean `hosp_admission`. Prior and shape of distribution informed by analysis of CO-CIN data[17].
`icu_admission`	Mean delay in days from start of infectious period to ICU admission	$\sim normal(12.5, 1)$, ≥ 8 and ≤ 14	Delay is assumed to follow a gamma distribution with shape parameter 1.91 and mean `icu_admission`. Prior and shape of distribution informed by analysis of CO-CIN data[17].
`f102, f144, f186, f228, f270, f312, f354, f396, f438, f480, f522, f564, f606, f648, f690, f732, f774, f816`	Contact multiplier for 6-week consecutive periods, with the first 6-week period starting from 12th April 2020 (f102) and the last 6-week period starting from 27th March 2022 (f816).	$\sim lognormal(0, 0.1)$, ≥ 0.5 and ≤ 2	Vague prior

Supplementary Table 5B. Model parameters not subject to fitting.

Parameter	Description	Value	Reference
d_E	Latent period (E to I_P and E to I_S; days)	$\sim gamma(\mu = 2.5, k = 2.5)$	Set to 2.5 so that incubation period (latent period plus period of preclinical infectiousness) is 5 days[18]
d_P	Duration of preclinical infectiousness (I_P to I_C; days)	$\sim gamma(\mu = 2.5, k = 4)$	Assumed to be half the duration of total infectiousness in clinically-infected individuals [19]
d_C	Duration of clinical infectiousness (I_C to R; days)	$\sim gamma(\mu = 2.5, k = 4)$	Infectious period set to 5 days, to result in a serial interval of approximately 6 days[20–22]
d_S	Duration of subclinical infectiousness (I_S to R; days)	$\sim gamma(\mu = 5.0, k = 4)$	Assumed to be the same duration as total infectious period for clinical cases, including preclinical transmission
y_i	Probability of clinical symptoms given infection for age group *i*	Estimated from case distributions across 6 countries	[23]
f	Relative infectiousness of subclinical cases	50%	Assumed[23,24]
c_{ij}	Number of age-*j* individuals contacted by an age-*i* individual per day, prior to changes in mobility	UK-specific contact matrix	[25]
N_i	Number of age-*i* individuals	From demographic data	[26]
Δt	Time step for discrete-time simulation	0.25 days	
$P(ICU)_i$	Proportion of hospitalised cases that require critical care for age group *i*	Estimated from CO-CIN data	[27]
`dVa1 / dVb1`	The duration that individuals have first-dose levels of vaccine protection before transitioning to second-dose levels. Note that we assume a 28-day delay between individuals receiving their first-dose and moving into first-dose levels of protection, and an equivalent 14-day delay before individuals reach second-dose efficacy. These duration assumptions take those delays into account.	Initially, $\sim gamma(\mu = 7.5, k = 1000)$<br>7.5 = 21.5 - 28 + 14<br>Then,<br>$\sim gamma(\mu = 57.8, k = 1000)$<br>57.8 = 71.8 - 28 +14	The average delay between first and second vaccine doses was 21.5 days prior to 26th January 2021 when the JCVI updated their guidance[28] on dosing schedules, extending the maximum recommended dosing gap. Following this, the average delay was measured as more than 71 days, using data from October 2021.
`dVa2 / dVb2`	The duration that individuals have second-dose levels of vaccine protection before receiving a booster vaccine or transitioning to a second-dose + waned state. Note that we assume a 14-day delay before individuals reach second-dose efficacy. These duration assumptions take those delays into account.	$\sim gamma(\mu = 205, k = 2000)$<br>290 days between 8th December 2020 and 24th September 2021<br>28 + (71 - 14) = 85 = average d1:d2 delay<br>290 - 85 = 205 days	The JCVI initially recommended a 6-month delay between second doses and booster vaccinations. Vaccine rollout began on 8th December 2020 and booster rollout began in September 2021.
`P(boost)`	The age-specific probability of receiving a COVID-19 booster vaccination, given primary vaccination with either a viral-vector or an mRNA	PBOOST=c(0, 0, 0, 0.4, 0.544, 0.597, 0.643, 0.698, 0.757, 0.809, 0.861, 0.892, 0.917, 0.946, 0.964, 0.967)	These probabilities are chosen by referring to NHS England data on monthly COVID-19 vaccinations, published on

	vaccine. Probabilities are given in order for each 5-year age group in the model, from 0-4 years up to 70-74 years, and finally for individuals aged 75 years and above.		14th April 2022[10]. We refer to the number of individuals who have received a booster or third vaccine divided by the number of individuals who have received a second vaccine, rounding to 3 decimal places, to inform our assumptions. For the 75+ model age group, we take the average of measured uptake for age groups 75-79 and 80+ in the NHS England data. We assume that individuals aged 16 years and above receive booster vaccinations and that by April 2022, maximum uptake levels have been reached for individuals aged 20 years and above (since these age groups have been eligible to receive boosters since December 2021). For individuals aged 16-19 in the 15-19 years age group, we assume booster uptake will reach 40% across the whole age group, referencing the different levels of uptake already achieved for older age groups (e.g. we calculate 20-24 year olds as having approximately 54% of booster dose uptake in eligible individuals, the lowest level of uptake across all age groups in the NHS England data).

Supplementary Table 6 - Vaccine effectiveness against pre-B.1.1.7 and Alpha B.1.1.7 variants - relevant evidence and basecase model assumptions

Description	Relevant evidence, assumed value shown in bold
Overall protection against infection for AstraZeneca dose 1	Shrotri et al. Table 4 adjusted hazard ratio 0.33 (0.16, 0.68) at 28-34 days post vaccination for protection against infection in care home residents. Pritchard et al., supplementary information, Table 6, adjusted odds ratio >= 21 days after first dose of AZ, no second dose, 0.39 (0.32, 0.46) for all positives. Glampson et al. results, Table 2, hazard ratio 0.26 (0.19, 0.35) for AZ between 22 and 28 days following first dose when comparing AZ vaccinated individuals with unvaccinated individuals. Thus a 74% reduction in risk of testing positive for COVID-19. Amirthalingam et al. results, adults aged 80 and above, 43% vaccine protection (24-58%) on days 28-34 following the first dose of AZ. Amongst 65-79 year olds 55% vaccine protection (48-61%) 28 days after the first dose of AZ. Amongst 50-64 year olds 50% vaccine protection (45-55%) 28 days after the first dose of AZ. Evidence of reductions in vaccine protection by 70 days post vaccination: 40% (23-53%) and 26% (18-33%) for 65-79 and 50-64 year olds respectively. Point estimates showed a decline after 8 weeks for individuals aged 80 and above (but wide confidence intervals). Pouwels et al. report vaccine effectiveness against all PCR-confirmed infections with the Alpha variant of 63% (55-69%) at least 21 days following the first dose of AZ. Sheikh et al. report vaccine effectiveness against PCR-confirmed infection (regardless of symptom status) of 37% (32-42%) 28 days after the first dose of AZ.<br><br>**0.7 (+28 days)**
Overall protection against disease for AstraZeneca dose 1	Pritchard et al., supplementary information, Table 6, adjusted odds ratio >= 21 days after first dose of AZ, no second dose, 0.29 (0.22, 0.38) for positive individuals with symptoms reported. Lopez Bernal et al. (cohort aged 70+ years of age) Table 3, ChAdOx1 adjusted odds ratio d1:28-34 0.4 (0.27-0.59). PHE's week 20 vaccine surveillance reports reports estimates of 53% (49-57%) vaccine protection against symptomatic disease at least 28 days following the first dose of AZ (compared to unvaccinated individuals). Compared to individuals between 4 and 13 days after the first dose, they estimate 58% (54-62%) protection against symptomatic disease. Whitaker et al. Table 4, adjusted vaccine effectiveness against symptomatic COVID-19 28-90 days post first dose of AZ 50.2% (40.8-58.2%) for individuals aged 16-64 and 60.9% (49.0-70.0%) for individuals aged 65 and over. Lopez Bernal et al. C report adjusted vaccine effectiveness against symptomatic infection with S-gene target negatives (i.e. Alpha variant) of 48.7% (45.2% to 51.9%). Pouwels et al. report vaccine effectiveness against symptomatic PCR-confirmed infections with the Alpha variant of 73% (67-77%) at least 21 days following the first dose of AZ. Sheikh et al. report vaccine effectiveness against symptomatic PCR-confirmed infection of 39% (32-45%) 28 days after the first dose of AZ. PHE's week 36 vaccine surveillance report estimates protection against symptomatic disease of 49% (46-52%) for the Alpha variant at least 28 days after a first vaccine dose. Andrews et al. report protection against symptomatic disease for the first dose of AZ as 44.5% (42.9 to 46.1%), at least 28 days following the first dose and up to the second dose if given.<br><br>**0.7 (+28 days) as for infection**
Overall protection against hospitalisation for AstraZeneca dose 1	Lopez Bernal et al. Table 4, hazard ratio for risk of hospital admission in vaccinated vs unvaccinated individuals (subsection of cohort that are 80+ years of age) 0.63 (0.41 to 0.97) at least 14 days following first dose of AZ. Vasileiou et al. Table 2, vaccine programme effect for ChAdOx1 21-27 days post first vaccine is 81% (72 to 87%), 28-34 days post first vaccine is 88% (75-94%), 35-41 days post first vaccine is 97% (63-100%). Smaller numbers. Table 3 splits analysis into age groups for ChAdOx1. Ismail et al. estimate vaccine effectiveness against hospitalisation of 73% (60-81%) for

	80+ year olds and 84% (74-89%) for 70-79 year olds, 28 days following the first dose of AZ. When analysis is not split across vaccine products, the same study estimates efficacy against hospitalisation of 80% (74-85%) for 80+ year olds and 82% (75-87%) for 70-79 year olds. Hyams et al. Table 2, adjusted vaccine effectiveness against hospitalisation (in individuals aged over 80 years of age) for one dose of ChAdOx1 nCov-19 80.4% (36.4 - 94.5%). PHE's week 26 vaccine surveillance report finds protection against hospitalisation with the Alpha variant of 79% (74-82%) following the first dose of AZ. Stowe et al. report vaccine effectiveness against hospitalisation of 76% (61-85%) following the first dose of AZ. PHE's week 36 vaccine surveillance report estimates protection against hospitalisation of 78% (64-87%) for the Alpha variant at least 28 days after a first vaccine dose. Andrews et al. report protection against hospitalisation for the first dose of AZ as 82.5% (78.7 to 85.7%), at least 28 days following the first dose and up to the second dose if given.<br><br>**0.85 (+28 days)**
Overall protection against mortality for AstraZeneca dose 1	Lopez Bernal et al. B (study in a care home population) estimated a hazard ratio of 0.45 (0.34 - 0.59) for cases vaccinated with one dose of AZ compared to unvaccinated cases, indicating an additional 55% (41-66%) protection against death given becoming a case for individuals vaccinated with one dose of AZ. Using the aforementioned estimate of a 55% increase and assuming this in addition to protection against disease of 0.7, we get overall protection against mortality of 86.5%. PHE's week 26 vaccine surveillance report finds protection against mortality with the Alpha variant of 79% (73-83%) and 83% (78-86%) for 40-64 and 65+ year olds respectively, following the first dose of AZ. Andrews et al. report protection against death for the first dose of AZ as 79.1% (68.8 to 86%), at least 28 days following the first dose and up to the second dose if given.<br><br>**0.85 (+28 days)**
Overall protection against onward transmission for AstraZeneca dose 1	Harris et al. calculate an adjusted odds ratio of being a secondary case within a household of index cases vaccinated with ChAdOx1 (AstraZeneca) at least 21 days before testing positive as 0.52 (0.43-0.62) and index cases vaccinated with BNT162b2 (Pfizer-BioNTech) at least 21 days before testing positive as 0.54 (0.47-0.62). Shah et al. find that relative to the period before a healthcare worker was vaccinated, the hazard ratio for a household member of the vaccinated healthcare worker to become infected was 0.7 (0.63-0.78) for the period beginning 14 days following first vaccine dose and 0.46 (0.30-0.70) for the period beginning 14 days after the second vaccine dose (healthcare workers were vaccinated with either AstraZeneca or Pfizer). Braeye et al. (Belgium, mostly Alpha variant) estimated VE against onward transmission "at 62% (95% CI 57–67) for BNT162b2 and 52% (95% CI 33–69) for mRNA1273 for full vaccination. No significant effect against onward transmission was found for the 'viral-vector'-vaccines, but credibility intervals were large." Eyre et al. report an adjusted odds ratio of 0.82 (0.76, 0.88) for the effect of a case being partially vaccinated with AZ (dose 1 day 1 to dose 2 +14 days) compared to an unvaccinated case in relation to the likelihood of a contact testing PCR-positive.<br><br>**0.47 (+28 days)**
Overall protection against infection for AstraZeneca dose 2	Shrotri et al. Table 4 adjusted hazard ratio 0.32 (0.15, 0.66) at 35-48 days post vaccination in care home residents. Pritchard et al., supplementary information, Table 6, adjusted odds ratio post second dose of AZ 0.21 (0.12, 0.35) for all positives. Lopez Bernal et al. (cohort aged 70+ years of age) Table 3, ChAdOx1 adjusted odds ratio d1:>=35 days 0.27 (0.10 to 0.73). Amirthalingam et al. results, individuals aged 80+ years old had 96% (68-99%) and 82% (68-89%) vaccine effect at least 14 days following the second dose of AZ with 45-64 and 65-84 day intervals between first and

	second doses. "Those receiving their second dose outside of these recommended intervals also had high VE after two doses; for an ≥85 day interval, the estimated VE was 88% (95%CI: 48-97)." Pouwels et al. report vaccine effectiveness against all PCR-confirmed infections with the Alpha variant of 79% (56-90%) at least 14 days following the second dose of AZ. Sheikh et al. report vaccine effectiveness against PCR-confirmed infection (regardless of symptom status) of 73% (66-78%) 14 days after the second dose of AZ.<br><br>**0.75 (+14 days)**
Overall protection against disease for AstraZeneca dose 2	Pritchard et al., supplementary information, Table 6, adjusted odds ratio post second dose of AZ 0.08 (0.03, 0.22) for positive individuals with symptoms reported. Voysey et al. A randomised controlled trial for ChAdOx1 nCoV-19 vaccine AZD1222, Table 3, average of efficacies more than 14 days after a second dose for LD/SD and SD/SD in 'COV002 (UK), age 18–55 years with >8 weeks' interval between vaccine doses*' row -> 0.778 = (0.9+0.656)/2. PHE's week 20 vaccine surveillance report reports estimates of 89% (78-94%) vaccine protection against symptomatic disease at least 14 days following the second dose of AZ (compared to unvaccinated individuals). Compared to individuals between 4 and 13 days post first dose, they estimate 90% (80-95%) protection. Whitaker et al. Table 4, adjusted vaccine effectiveness against symptomatic COVID-19 at least 14 days following second dose of AZ 78% (69.7-84%) for individuals aged 16-64 and 76.4% (58.8-86.5%) for individuals aged 65 and over. Lopez Bernal et al. C report adjusted vaccine effectiveness against symptomatic infection with S-gene target negatives (Alpha variant) of 74.5% (68.4% to 79.4%) at least 14 days after the second dose of AZ. Pouwels et al. report vaccine effectiveness against symptomatic PCR-confirmed infections with the Alpha variant of 97% (93-98%) at least 14 days following the second dose of AZ. Sheikh et al. report vaccine effectiveness against symptomatic PCR-confirmed infection of 81% (72-87%) 14 days after the second dose of AZ. PHE's week 36 vaccine surveillance report estimates protection against symptomatic disease of 89% (87-90%) for the Alpha variant at least 14 days after a second vaccine dose. Andrews et al. report protection against symptomatic disease for the second dose of AZ as 81.7% (79.0 to 84.0%), at least 14 days following the second dose.<br><br>**0.8 (+14 days)**
Overall protection against hospitalisation for AstraZeneca dose 2	Ismail et al. estimate vaccine effectiveness against hospitalisation of 92% (87-95%) 14 days after a second dose across both AZ and Pfizer vaccines. PHE's week 26 vaccine surveillance report finds protection against hospitalisation with the Alpha variant of 94% (81-98) following the second dose of AZ. Stowe et al. report vaccine effectiveness against hospitalisation of 86% (53-96%) following the second dose of AZ. PHE's week 36 vaccine surveillance report estimates protection against hospitalisation of 93% (80-97%) for the Alpha variant at least 14 days after a second vaccine dose. Andrews et al. report protection against hospitalisation for the second dose of AZ as 93.9% (84.9 to 97.5%), at least 14 days following the second dose.<br><br>**0.9 (+14 days)**
Overall protection against mortality for AstraZeneca dose 2	PHE's week 26 vaccine surveillance report finds protection against mortality with the Alpha variant of 92% (76-98%) and 94% (80-98%) for 40-64 and 65+ year olds respectively, following the second dose of AZ. Andrews et al. report protection against death for the second dose of AZ as 100% at least 14 days following the second dose.<br><br>**0.95 (+14 days)**
Overall protection against onward transmission for AstraZeneca dose	Shah et al. find that relative to the period before a healthcare worker was vaccinated, the hazard ratio for a household member of the vaccinated

2	healthcare worker to become infected was 0.7 (0.63-0.78) for the period beginning 14 days following first vaccine dose and 0.46 (0.30-0.70) for the period beginning 14 days after the second vaccine dose (healthcare workers were vaccinated with either AstraZeneca or Pfizer). Braeye et al. (Belgium, mostly Alpha variant) estimated VE against onward transmission “at 62% (95% CI 57–67) for BNT162b2 and 52% (95% CI 33–69) for mRNA1273 for full vaccination. No significant effect against onward transmission was found for the ‘viral-vector’-vaccines, but credibility intervals were large.” Eyre et al. report an adjusted odds ratio of 0.37 (0.22, 0.63) for the effect of a case being fully vaccinated with AZ (dose 2 +14 days) compared to an unvaccinated case in relation to the likelihood of a contact testing PCR-positive.<br><br>**0.47 (+14 days)**
Overall protection against infection for Pfizer dose 1	Hall et al. Table 2, full cohort adjusted hazard ratio d1>=21 days 0.30 (0.15-0.45). Pritchard et al. supplementary information, Table 6, adjusted odds ratio >= 21 days after first dose of Pfizer, no second dose, 0.34 (0.29, 0.40) for all positives. Shrotri et al. Table 4 adjusted hazard ratio 0.47 (0.20, 1.06) at 28-34 days post vaccination for protection against infection in care home residents. Glampson et al. results, Table 2, hazard ratio 0.22 (0.18, 0.27) for Pfizer between 22 and 28 days following first dose when comparing Pfizer vaccinated individuals with unvaccinated individuals. Thus a 78% reduction in risk of testing positive for COVID-19. Mason et al. Table 2, vaccine effect of 55.2% (40.8 - 66.8%) 21-27 days post first dose, of 53.7% (35.4 - 66.6%) 28-34 days post first dose and of 70.1% (55.1 - 80.1%) 35-41 days post first dose in individuals aged 80-83 years of age. Azamgarhi et al. Table 2, 14 days after first vaccination dose in healthcare workers find an adjusted hazard ratio of 0.3 (0.09,0.94) for protection against documented infection. Amirthalingam et al. results, 80+ year olds had 61% (49-71%) vaccine protection with a 3-week dosing schedule at 28-34 days post first dose of Pfizer. 80+ year olds with the longer dosing interval had 52% (39-63%) vaccine protection 28-34 days following the first dose of Pfizer. “Amongst 65-79 year-olds, VE began to increase from 10-13 days after vaccination, reaching 53% (95%CI: 45-60) on days 28-34, and remained at a similar level between 35-69 days (5-10 weeks). A similar trend was observed in the BNT162b2 recipients aged 50- 64 years with a VE of 58% at days 28-34. Whilst there was some evidence of a 10-20% decrease in VE by 10 weeks after the first dose, there was an apparent rise again in VE at the final interval, although with wide confidence intervals”. Pouwels et al. report vaccine effectiveness against all PCR-confirmed infections with the Alpha variant of 59% (52-65%) at least 21 days following the first dose of Pfizer. Sheikh et al. report vaccine effectiveness against PCR-confirmed infection (regardless of symptom status) of 38% (29-45%) 28 days after the first dose of Pfizer.<br><br>**0.7 (+28 days)**
Overall protection against disease for Pfizer dose 1	Lopez Bernal et al. (cohort aged 70+ years of age) Table 2, odds ratio vs day 4-9, d1:28-34 0.30 (0.22-0.41). Pritchard et al., supplementary information, Table 6, adjusted odds ratio >= 21 days after first dose of Pfizer, no second dose, 0.22 (0.17, 0.28) for positive individuals with symptoms reported. PHE’s week 20 vaccine surveillance report estimates protection against symptomatic disease at least 28 days following the first dose of Pfizer as 54% (50-58%) compared to unvaccinated individuals. Compared to individuals between 4 and 13 days post first dose, they estimate 57% (53-61%) protection. Whitaker et al. Table 4, adjusted vaccine effectiveness against symptomatic COVID-19 28-90 days post first dose of Pfizer 48.6% (27.9-63.3%) for individuals aged 16-64 and 56.6% (47.6-64.1%) for individuals aged 65 and over. Lopez Bernal et al. C report adjusted vaccine effectiveness against symptomatic infection with S-gene target negatives (i.e. Alpha variant) of 47.5% (41.6% to 52.8%). Pouwels et al. report vaccine effectiveness against symptomatic PCR-confirmed infections with the Alpha variant of 73% (68-76%) at least 21 days following the first dose of Pfizer.

	Sheikh et al. report vaccine effectiveness against symptomatic PCR-confirmed infection of 27% (13-39%) 28 days after the first dose of Pfizer. PHE's week 36 vaccine surveillance report estimates protection against symptomatic disease of 49% (46-52%) for the Alpha variant at least 28 days after a first vaccine dose. Andrews et al. report protection against symptomatic disease for the first dose of Pfizer as 45.7% (44 to 47.3%), at least 28 days following the first dose and up to the second dose if given.<br><br>**0.7 (+28 days)**
Overall protection against hospitalisation for Pfizer dose 1	Lopez Bernal et al. Table 4, hazard ratio for risk of hospital admission in vaccinated vs unvaccinated individuals (subsection of cohort that are 80+ years of age) 0.57 (0.48 to 0.67) at least 14 days following first dose of Pfizer. Hyams et al. Table 2, adjusted vaccine effectiveness (in individuals aged 80 years and above) for one dose of BNT162b2 71.4% (43.1 - 86.2%). When the analysis of the effectiveness of one dose of BNT162b2 was restricted to the period covered by the ChAdOx1nCoV-19 analysis after the end of 2020, the observed adjusted estimate was 79.3% (95% CI 47.0-92.5) (P=0.0014). Dagan et al. estimate vaccine effectiveness against hospitalisation of 74% (56–86%) 14-20 days after first dose and 78% (61–91%) 21 to 27 days after first dose. Vasileiou et al. Table 2, vaccine effect for BNT162b2 21-27 days post first vaccine is 78% (71 to 83) and 28-34 days post first vaccine is 91% (85 to 94). Estimated vaccine effect against hospitalisation is reduced for later time points to 78% and 77%. Table 3 split vaccine effect into age groups. Ismail et al. estimate vaccine effectiveness against hospitalisation of 81% (76-85%) for 80+ year olds and 81% (73-87%) for 70-79 year olds, 28 days following the first dose of Pfizer. When the analysis is not split across vaccine products, the same study estimates protection against hospitalisation of 80% (74-85%) for 80+ year olds and 82% (75-87%) for 70-79 year olds, 28 days following the first vaccine dose. Mason et al. Table 2, vaccine effect against hospital admission of 50.1% (19.9 - 69.5%) 21-27 days post first dose, of 63.7% (37.1 - 79.2%) 28-34 days post first dose and of 75.6% (52.8 - 87.6%) 35-41 days post first dose in individuals aged 80-83 years of age. Vaccine effect against A&E (accident & emergency) hospital attendance of 57.8% (30.8 - 74.5%) 21-27 days post first dose, of 68.1% (45.2 - 80.9%) 28-34 days post first dose and of 78.9% (60.0 - 89.9%) 35-41 days post first dose in individuals aged 80-83 years of age. PHE's week 26 vaccine surveillance report finds protection against hospitalisation with the Alpha variant of 82% (78-85%) following the first dose of Pfizer. Stowe et al. report vaccine effectiveness against hospitalisation of 83% (62-93%) following the first dose of Pfizer. PHE's week 36 vaccine surveillance report estimates protection against hospitalisation of 78% (64-87%) for the Alpha variant at least 28 days after a first vaccine dose. Andrews et al. report protection against hospitalisation for the first dose of Pfizer as 85.2% (81.6 to 88.1%), at least 28 days following the first dose and up to the second dose if given.<br><br>**0.85 (+28 days)**
Overall protection against mortality for Pfizer dose 1	Dagan et al. estimate vaccine effectiveness against mortality of 72% (19–100%) 14-20 days after first dose and 84% (44–100%) 21 to 27 days after first dose. Lopez Bernal et al. B (study in a care home population) estimated a hazard ratio of 0.56 (0.47 - 0.68) for cases vaccinated with one dose of Pfizer compared to unvaccinated cases, indicating an additional 44% (32-53%) protection against death given becoming a case for individuals vaccinated with one dose of Pfizer. Using the aforementioned estimate of a 44% increase and assuming this in addition to protection against disease of 0.7, we get overall protection against mortality of 0.832. PHE's week 26 vaccine surveillance report finds protection against mortality with the Alpha variant of 73% (67-77%) and 77% (72-81%) for 40-64 and 65+ year olds respectively, following the first dose of Pfizer. Andrews et al. report protection against death for the first dose of Pfizer as 73.1% (65 to 79.3%), at least 28 days following the first dose and up to the second dose if given.

	**0.85 (+28 days)**
Overall protection against onward transmission for Pfizer dose 1	Harris et al. calculate an adjusted odds ratio of being a secondary case within a household of index cases vaccinated with ChAdOx1 (AstraZeneca) at least 21 days before testing positive as 0.52 (0.43-0.62) and index cases vaccinated with BNT162b2 (Pfizer-BioNTech) at least 21 days before testing positive as 0.54 (0.47-0.62). Shah et al. find that relative to the period before a healthcare worker was vaccinated, the hazard ratio for a household member of the vaccinated healthcare worker to become infected was 0.7 (0.63-0.78) for the period beginning 14 days following first vaccine dose and 0.46 (0.30-0.70) for the period beginning 14 days after the second vaccine dose (healthcare workers were vaccinated with either AstraZeneca or Pfizer). Braeye et al. (Belgium, mostly Alpha variant) estimated VE against onward transmission “at 62% (95% CI 57–67) for BNT162b2 and 52% (95% CI 33–69) for mRNA1273 for full vaccination. No significant effect against onward transmission was found for the ‘viral-vector’-vaccines, but credibility intervals were large.” Eyre et al. report an adjusted odds ratio of 0.74 (0.70, 0.80) for the effect of a case being partially vaccinated with Pfizer (dose 1 day 1 to dose 2 +14 days) compared to an unvaccinated case in relation to the likelihood of a contact testing PCR-positive.<br><br>**0.47 (+28 days)**
Overall protection against infection for Pfizer dose 2	Hall et al. Table 2, full cohort adjusted hazard ratio d2>=7 days 0.15 (0.04-0.26). Pritchard et al., supplementary information, Table 6, adjusted odds ratio post second dose of Pfizer 0.20 (0.15, 0.26) for all positives. Haas et al. estimate vaccine protection against SARS-CoV-2 infection (both asymptomatic and symptomatic and symptoms unknown) of 95.3% (94.9-95.7%). Shrotri et al. Table 4 adjusted hazard ratio 0.35 (0.17, 0.71) at 35-48 days post vaccination (first dose) for protection against infection in care home residents, but no estimates related to second vaccine dose. Pouwels et al. report vaccine effectiveness against all PCR-confirmed infections with the Alpha variant of 78% (68-84%) at least 14 days following the second dose of Pfizer. Sheikh et al. report vaccine effectiveness against PCR-confirmed infection (regardless of symptom status) of 92% (90-93%) 14 days after the second dose of Pfizer.<br><br>**0.85 (+14 days)**
Overall protection against disease for Pfizer dose 2	Lopez Bernal et al. (cohort aged 70+ years of age) Table 2, odds ratio vs day 4-9, d2:14+ 0.11 (0.07-0.15). Haas et al. estimate vaccine protection against symptomatic COVID-19 >7 days after second dose of 97% (96.7-97.2%). Pritchard et al., supplementary information, Table 6, adjusted odds ratio post second dose of Pfizer 0.05 (0.02, 0.09) for positive individuals with symptoms reported. PHE’s week 20 vaccine surveillance report estimates protection against symptomatic disease at least 14 days following the second dose of Pfizer as 90% (82-95%) compared to unvaccinated individuals. Compared to individuals between 4 and 13 days post first dose, they estimate 91% (83-95%) protection. Whitaker et al. Table 4, adjusted vaccine effectiveness against symptomatic COVID-19 at least 14 days following the second dose of Pfizer 93.3% (85.8-96.8%) for individuals aged 16-64 and 86.7% (80.1-91.1%) for individuals aged 65 and over. Pouwels et al. report vaccine effectiveness against symptomatic PCR-confirmed infections with the Alpha variant of 97% (96-98%) at least 14 days following the second dose of Pfizer. Sheikh et al. report vaccine effectiveness against symptomatic PCR-confirmed infection of 92% (88-94%) 14 days after the second dose of Pfizer. PHE’s week 36 vaccine surveillance report estimates protection against symptomatic disease of 89% (87-90%) for the Alpha variant at least 14 days after a second vaccine dose. Andrews et al. report protection against symptomatic disease for the second dose of Pfizer as 95.0% (93.8 to 95.9%) at least 14 days following the second dose.<br><br>**0.9 (+14 days)**

Overall protection against hospitalisation for Pfizer dose 2	Dagan et al. estimate vaccine effectiveness against hospitalisation of 87% (55–100%) >7 days after second dose. Haas et al. estimate vaccine protection against COVID-19 related hospitalisation >7 days after second dose of 97.2% (96.8-97.5%). Ismail et al. estimates vaccine protection against hospitalisation of 93% (89-95%) for individuals aged 80+ years 14 days after receiving their second dose of Pfizer. When the analysis is not split by vaccine type, the same study estimates protection against hospitalisation of 92% (87-95%) for 80+ year olds 14 days after second dose. PHE's week 26 vaccine surveillance report finds protection against hospitalisation with the Alpha variant of 98% (96-99%) following the second dose of Pfizer. Stowe et al. report vaccine effectiveness against hospitalisation of 95% (78-99%) following the second dose of Pfizer. PHE's week 36 vaccine surveillance report estimates protection against hospitalisation of 93% (80-97%) for the Alpha variant at least 14 days after a second vaccine dose. Andrews et al. report protection against hospitalisation for the second dose of Pfizer as 97.9% (91.4 to 99.5%) at least 14 days following the second dose.<br><br>**0.95 (+14 days)**
Overall protection against mortality for Pfizer dose 2	Dagan et al. estimate vaccine effectiveness against mortality of 72% (19–100%) 14-20 days after first dose and 84% (44–100%) 21 to 27 days after first dose (no estimates for second dose protection). The same study estimates protection against severe disease of 92% (75-100%) >7 days following the second dose of Pfizer. Haas et al. estimate vaccine protection against death >7 days after second dose of 96.7% (96.0-97.3%). Lopez Bernal et al. B (study in a care home population) estimated a hazard ratio of 0.31 (0.14 - 0.69) for cases vaccinated with two doses of Pfizer compared to unvaccinated cases, indicating an additional 69% (31-86%) protection against death given becoming a case for individuals vaccinated with two doses of Pfizer. Using the aforementioned estimate of a 69% increase and assuming this in addition to protection against disease of 0.9**,** we get overall protection against mortality of 96.9%**.** PHE's week 26 vaccine surveillance report finds protection against mortality with the Alpha variant of 98% (94-99%) for both 40-64 and 65+ year olds following the second dose of Pfizer. Andrews et al. report protection against death for the second dose of Pfizer as 96.3% (89.9 to 98.6%) at least 14 days following the second dose.<br><br>**0.95 (+14 days)**
Overall protection against onward transmission for Pfizer dose 2	Shah et al. find that relative to the period before a healthcare worker was vaccinated, the hazard ratio for a household member of the vaccinated healthcare worker to become infected was 0.7 (0.63-0.78) for the period beginning 14 days following first vaccine dose and 0.46 (0.30-0.70) for the period beginning 14 days after the second vaccine dose (healthcare workers were vaccinated with either AstraZeneca or Pfizer). Braeye et al. (Belgium, mostly Alpha variant) estimated VE against onward transmission "at 62% (95% CI 57–67) for BNT162b2 and 52% (95% CI 33–69) for mRNA1273 for full vaccination. No significant effect against onward transmission was found for the 'viral-vector'-vaccines, but credibility intervals were large." Eyre et al. report an adjusted odds ratio of 0.18 (0.12, 0.29) for the effect of a case being fully vaccinated with Pfizer (dose 2 +14 days) compared to an unvaccinated case in relation to the likelihood of a contact testing PCR-positive.<br><br>**0.47 (+14 days)**

Supplementary Table 7 - Vaccine effectiveness against B.1.617.2 Delta variant - relevant evidence and baseline model assumptions

Description	Relevant evidence, assumed value shown in bold
Overall protection against infection for AstraZeneca dose 1	Pouwels et al. report vaccine effectiveness against all PCR-confirmed infections with the Delta variant of 46% (35-55%) at least 21 days following the first dose of AZ. This is a **26.98% reduction** on their equivalent estimate for the Alpha variant. Sheikh et al. report vaccine effectiveness against PCR-confirmed infection (regardless of symptom status) of 18% (9-25%) 28 days after the first dose of AZ. This is a **51.35% reduction** on their equivalent estimate for the Alpha variant.<br><br>**Alpha assumption 0.7**<br><br>**0.43 = 0.7 * (1 - 0.39) (+28 days)**
Overall protection against disease for AstraZeneca dose 1	Lopez Bernal et al. C report adjusted vaccine effectiveness against symptomatic infection with S-gene target positives (Delta variant) of 30.0% (24.3-35.3%) at least 21 days after the first dose of AZ. A **38.4% reduction** on their estimate of equivalent vaccine protection against the Alpha variant. Pouwels et al. report vaccine effectiveness against symptomatic PCR-confirmed infections with the Delta variant of 40% (28-50%) at least 21 days following the first dose of AZ. This is a **45.21% reduction** on their equivalent estimate for the Alpha variant. Sheikh et al. report vaccine effectiveness against symptomatic PCR-confirmed infection of 33% (23-41%) 28 days after the first dose of AZ. This is a **15.38% reduction** on their equivalent estimate for the Alpha variant. PHE's week 36 vaccine surveillance report estimates protection against symptomatic disease of 35% (32-38%) for the Delta variant at least 28 days after a first vaccine dose. This is a **28.57% reduction** compared to their equivalent estimate for Alpha. Andrews et al. report protection against symptomatic disease for the first dose of AZ as 43.3% (42.3 to 44.2%), at least 28 days following the first dose and up to the second dose if given. This is a **2.7% reduction** compared to their equivalent estimate for Alpha.<br><br>**Alpha assumption 0.7**<br><br>**0.52 = 0.7 * (1-0.26) (+28 days)**
Overall protection against hospitalisation for AstraZeneca dose 1	Stowe et al. report vaccine effectiveness against hospitalisation of 71% (51-83%) following the first dose of AZ. This is a **6.58% reduction** on their equivalent estimate for the Alpha variant. PHE's week 36 vaccine surveillance report estimates protection against hospitalisation of 80% (69-88%) for the Delta variant at least 28 days after a first vaccine dose. This is a **2.56% increase** compared to their equivalent estimate for Alpha. Andrews et al. report protection against hospitalisation for the first dose of AZ as 81.4% (78.7 to 83.7%), at least 28 days following the first dose and up to the second dose if given. This is a **1.3% reduction** compared to their equivalent estimate for Alpha.<br><br>**Alpha assumption 0.85**<br><br>**0.84 = 0.85 * (1-0.017) (+28 days)**
Overall protection against mortality for AstraZeneca dose 1	Andrews et al. report protection against death for the first dose of AZ as 88.4% (78.2 to 93.8%), at least 28 days following the first dose and up to the second dose if given. This is a **11.8% increase** compared to their equivalent

	estimate for Alpha.<br><br>**Alpha assumption 0.85**<br><br>**0.95 = 0.85 * (1 + 0.118) (+28 days)**
Overall protection against onward transmission for AstraZeneca dose 1	Eyre et al. report an adjusted odds ratio of 0.98 (0.90, 1.06) for the effect of a case being partially vaccinated with AZ (dose 1 day 1 to dose 2 +14 days) compared to an unvaccinated case in relation to the likelihood of a contact testing PCR-positive (N.B. this estimate has a non-significant p-value), equivalent to vaccine protection of 2%. Their equivalent estimate for Alpha is 0.82 (0.76, 0.88), therefore vaccine protection of 18%. This is a **88.9% overall reduction** in vaccine effect from Alpha to Delta.<br><br>**Alpha assumption 0.47**<br><br>**0.05 = 0.47 * (1 - 0.889) (+28 days)**
Overall protection against infection for AstraZeneca dose 2	Pouwels et al. report vaccine effectiveness against all PCR-confirmed infections with the Delta variant of 67% (62-71%) at least 14 days following the second dose of AZ. This is a **15.19% reduction** on their equivalent estimate for the Alpha variant. Sheikh et al. report vaccine effectiveness against PCR-confirmed infection (regardless of symptom status) of 60% (53-66%) 14 days after the second dose of AZ. This is a **17.81% reduction** on their equivalent estimate for the Alpha variant.<br><br>**Alpha assumption 0.75**<br><br>**0.63 = 0.75 * (1-0.165) (+14 days)**
Overall protection against disease for AstraZeneca dose 2	Lopez Bernal et al. C report adjusted vaccine effectiveness against symptomatic infection with S-gene target positives (Delta variant) of 67.0% (61.3% to 71.8%) at least 14 days after the second dose of AZ. A **10.07% reduction** on their estimate of equivalent vaccine protection against the Alpha variant. Pouwels et al. report vaccine effectiveness against symptomatic PCR-confirmed infections with the Delta variant of 71% (66-74%) at least 14 days following the second dose of AZ. This is a **26.8% reduction** on their equivalent estimate for the Alpha variant. Sheikh et al. report vaccine effectiveness against symptomatic PCR-confirmed infection of 61% (51-70%) 14 days after the second dose of AZ. This is a **24.69% reduction** on their equivalent estimate for the Alpha variant. PHE's week 36 vaccine surveillance report estimates protection against symptomatic disease of 79% (78-80%) for the Delta variant at least 14 days after a second vaccine dose. This is an **11.24% reduction** compared to their equivalent estimate for Alpha. Andrews et al. report protection against symptomatic disease for the second dose of AZ as 65.2% (64.9 to 65.6%), at least 14 days following the second dose. This is a **20.2% reduction** compared to their equivalent estimate for Alpha.<br><br>**Alpha assumption 0.8**<br><br>**0.65 = 0.8 * (1 - 0.186) (+14 days)**
Overall protection against hospitalisation for AstraZeneca dose 2	Stowe et al. report vaccine effectiveness against hospitalisation of 92% (75-97%) following the second dose of AZ. This is a **6.98% increase** on their equivalent estimate for the Alpha variant. PHE's week 36 vaccine surveillance report estimates protection against hospitalisation of 96% (91-98%) for the Delta variant at least 14 days after a second vaccine dose.

	This is a **3.23% increase** compared to their equivalent estimate for Alpha. Andrews et al. report protection against hospitalisation for the second dose of AZ as 93.0% (92.4 to 93.5%), at least 14 days following the second dose. This is a **1% reduction** compared to their equivalent estimate for Alpha.<br><br>**Alpha assumption 0.9**<br><br>**0.93 = 0.9 * (1 + 0.0307) (+14 days)**
Overall protection against mortality for AstraZeneca dose 2	Andrews et al. report protection against death for the second dose of AZ as 92.7% (90.7 to 94.3%), at least 14 days following the second dose. This is a **7.3% reduction** compared to their equivalent estimate for Alpha.<br><br>**Alpha assumption 0.95**<br><br>**0.95 (+14 days), as for dose 1 protection against mortality**
Overall protection against onward transmission for AstraZeneca dose 2	Eyre et al. report an adjusted odds ratio of 0.64 (0.57, 0.72) for the effect of a case being fully vaccinated with AZ (dose 2 +14 days) compared to an unvaccinated case in relation to the likelihood of a contact testing PCR-positive, equivalent to vaccine protection of 36%. Their equivalent estimate for Alpha is 0.37 (0.22, 0.63), therefore vaccine protection of 63%. This is a **42.9% overall reduction** in vaccine effect from Alpha to Delta.<br><br>**Alpha assumption 0.47**<br><br>**0.27 = 0.47 * (1 - 0.429) (+14 days)**
Overall protection against infection for Pfizer dose 1	Pouwels et al. report vaccine effectiveness against all PCR-confirmed infections with the Delta variant of 57% (50-63%) at least 21 days following the first dose of Pfizer. This is a **3.39% reduction** on their equivalent estimate for the Alpha variant. Sheikh et al. report vaccine effectiveness against PCR-confirmed infection (regardless of symptom status) of 30% (17-41%) 28 days after the first dose of Pfizer. This is a **21.05% reduction** on their equivalent estimate for the Alpha variant.<br><br>**Alpha assumption 0.7**<br><br>**0.62 = 0.7 * (1-0.12) (+28 days)**
Overall protection against disease for Pfizer dose 1	Lopez Bernal et al. C report adjusted vaccine effectiveness against symptomatic infection with S-gene target positives (Delta variant) of 35.6% (22.7% to 46.4%) at least 21 days after the first dose of Pfizer. A **25.05% reduction** on their estimate of equivalent vaccine protection against the Alpha variant. Pouwels et al. report vaccine effectiveness against symptomatic PCR-confirmed infections with the Delta variant of 58% (51-64%) at least 21 days following the first dose of Pfizer. This is a **20.55% reduction** on their equivalent estimate for the Alpha variant. Sheikh et al. report vaccine effectiveness against symptomatic PCR-confirmed infection of 33% (15-47%) 28 days after the first dose of Pfizer. This is a **22.22% reduction** on their equivalent estimate for the Alpha variant. PHE's week 36 vaccine surveillance report estimates protection against symptomatic disease of 35% (32-38%) for the Delta variant at least 28 days after a first vaccine dose. This is a **28.57% reduction** compared to their equivalent estimate for Alpha. Andrews et al. report protection against symptomatic disease for the first dose of Pfizer as 51.9% (51.4 to 52.4%), at least 28 days following the first dose and up to the second dose if given. This is a **13.6% increase** compared to their equivalent estimate for Alpha.

	**Alpha assumption 0.7**<br><br>**0.62 (+28 days) as for infection, otherwise would be 0.58**
Overall protection against hospitalisation for Pfizer dose 1	Stowe et al. report vaccine effectiveness against hospitalisation of 94% (46-99%) following the first dose of Pfizer. This is a **13.25% increase** on their equivalent estimate for the Alpha variant. PHE's week 36 vaccine surveillance report estimates protection against hospitalisation of 80% (69-88%) for the Delta variant at least 28 days after a first vaccine dose. This is a **2.56% increase** compared to their equivalent estimate for Alpha. Andrews et al. report protection against hospitalisation for the first dose of Pfizer as 91.8% (90.4 to 93%), at least 28 days following the first dose and up to the second dose if given. This is a **7.7% increase** compared to their equivalent estimate for Alpha.<br><br>**Alpha assumption 0.85**<br><br>**0.92 = 0.85 * (1+0.078) (+28 days)**
Overall protection against mortality for Pfizer dose 1	Andrews et al. report protection against death for the first dose of Pfizer as 88.6% (77.3 to 94.3%), at least 28 days following the first dose and up to the second dose if given. This is a **21.2% increase** compared to their equivalent estimate for Alpha.<br><br>**Alpha assumption 0.85**<br><br>**0.92 (+28 days) as for hospitalisation**
Overall protection against onward transmission for Pfizer dose 1	Eyre et al. report an adjusted odds ratio of 0.87 (0.81, 0.94) for the effect of a case being partially vaccinated with Pfizer (dose 1 day 1 to dose 2 +14 days) compared to an unvaccinated case in relation to the likelihood of a contact testing PCR-positive, equivalent to vaccine protection of 13%. Their equivalent estimate for Alpha is 0.74 (0.70, 0.80), therefore vaccine protection of 26%. This is a **50% overall reduction** in vaccine effect from Alpha to Delta.<br><br>**Alpha assumption 0.47**<br><br>**0.24 = 0.47 * (1 - 0.5) (+28 days)**
Overall protection against infection for Pfizer dose 2	Pouwels et al. report vaccine effectiveness against all PCR-confirmed infections with the Delta variant of 80% (77-83%) at least 14 days following the second dose of Pfizer. This is a **2.56% increase** on their equivalent estimate for the Alpha variant. Sheikh et al. report vaccine effectiveness against PCR-confirmed infection (regardless of symptom status) of 79% (75-82%) 14 days after the second dose of Pfizer. This is a **14.13% reduction** on their equivalent estimate for the Alpha variant.<br><br>**Alpha assumption 0.85**<br><br>**0.8 = 0.85 * (1-0.057) (+14 days)**

Overall protection against disease for Pfizer dose 2	Lopez Bernal et al. C report adjusted vaccine effectiveness against symptomatic infection with S-gene target positives (Delta variant) of 88.0% (85.3% to 90.1%) at least 14 days after the second dose of Pfizer. A **6.08% reduction** on their estimate of equivalent vaccine protection against the Alpha variant. Pouwels et al. report vaccine effectiveness against symptomatic PCR-confirmed infections with the Delta variant of 84% (82-86%) at least 14 days following the second dose of Pfizer. This is a **13.4% reduction** on their equivalent estimate for the Alpha variant. Sheikh et al. report vaccine effectiveness against symptomatic PCR-confirmed infection of 83% (78-87%) 14 days after the second dose of Pfizer. This is a **9.78% reduction** on their equivalent estimate for the Alpha variant. PHE's week 36 vaccine surveillance report estimates protection against symptomatic disease of 79% (78-80%) for the Delta variant at least 14 days after a second vaccine dose. This is an **11.24% reduction** compared to their equivalent estimate for Alpha. Andrews et al. report protection against symptomatic disease for the second dose of Pfizer as 83.5% (83.3 to 83.6%), at least 14 days following the second dose. This is a **12.1% reduction** compared to their equivalent estimate for Alpha.<br><br>**Alpha assumption 0.9**<br><br>**0.81 = 0.9 * (1 - 0.105) (+14 days)**
Overall protection against hospitalisation for Pfizer dose 2	Stowe et al. report vaccine effectiveness against hospitalisation of 96% (86-99%) following the second dose of Pfizer. This is a **1.05% increase** on their equivalent estimate for the Alpha variant. PHE's week 36 vaccine surveillance report estimates protection against hospitalisation of 96% (91-98%) for the Delta variant at least 14 days after a second vaccine dose. This is a **3.23% increase** compared to their equivalent estimate for Alpha. Andrews et al. report protection against hospitalisation for the second dose of Pfizer as 96.7% (96.3 to 97%), at least 14 days following the second dose. This is a **1.2% reduction** compared to their equivalent estimate for Alpha.<br><br>**Alpha assumption 0.95**<br><br>**0.96 = 0.95 * (1 + 0.0103) (+14 days)**
Overall protection against mortality for Pfizer dose 2	Andrews et al. report protection against death for the second dose of Pfizer as 95.2% (93.7 to 96.4%), at least 14 days following the second dose. This is a **1.1% reduction** compared to their equivalent estimate for Alpha.<br><br>**Alpha assumption 0.95**<br><br>**0.96 (+14 days) as for hospitalisation**
Overall protection against onward transmission for Pfizer dose 2	Eyre et al. report an adjusted odds ratio of 0.35 (0.26, 0.48) for the effect of a case being fully vaccinated with Pfizer (dose 2 +14 days) compared to an unvaccinated case in relation to the likelihood of a contact testing PCR-positive, equivalent to vaccine protection of 65%. Their equivalent estimate for Alpha is 0.18 (0.12, 0.29), therefore vaccine protection of 82%. This is a **20.7% overall reduction** in vaccine effect from Alpha to Delta.<br><br>**Alpha assumption 0.47**<br><br>**0.37 = 0.47 * (1 - 0.207) (+14 days)**

CMMID COVID-19 working group funding statements

The following funding sources are acknowledged as providing funding for the working group authors. PK (Royal Society: RP\EA\180004, European Commission: 101003688), TJ (Global Challenges Research Fund: ES/P010873/1, UK Public Health Rapid Support Team, NIHR: Health Protection Research Unit for Modelling Methodology HPRU-2012-10096, UK MRC: MC_PC_19065), AG (European Commission: 101003688), SFlasche (Wellcome Trust: 208812/Z/17/Z), RL (Royal Society: Dorothy Hodgkin Fellowship), DH (NIHR: 1R01AI141534-01A1), AJK (Wellcome Trust: 206250/Z/17/Z, NIHR: NIHR200908), HPG (EDCTP2: RIA2020EF-2983-CSIGN, UK DHSC/UK Aid/NIHR: PR-OD-1017-20001), SFunk (Wellcome Trust: 210758/Z/18/Z), GMK (UK MRC: MR/P014658/1), WW (MRC: MR/V027956/1), RME (HDR UK: MR/S003975/1, UK MRC: MC_PC_19065, NIHR: NIHR200908), MK (Wellcome Trust: 221303/Z/20/Z), LACC (NIHR: NIHR200908), YL (B&MGF: INV-003174, NIHR: 16/137/109, European Commission: 101003688, UK MRC: MC_PC_19065), KA (BMGF: INV-016832; OPP1157270), KO'R (B&MGF: OPP1191821), KEA (ERC: SG 757688), FYS (NIHR: 16/137/109), FGS (NIHR: NIHR200929), RP (Singapore Ministry of Health), PM CADDE (MR/S0195/1 & FAPESP 18/14389-0), SC (Wellcome Trust: 208812/Z/17/Z, UK MRC: MC_PC_19065), SA (Wellcome Trust: 210758/Z/18/Z), CIJ (Global Challenges Research Fund: ES/P010873/1), CVM (NIHR: NIHR200929), NIB (HPRU: NIHR200908), SRP (B&MGF: INV-016832), KLM (European Commission: 101003688), JH (Wellcome Trust: 210758/Z/18/Z), YJ (UKRI: MR/V028456/1), MQ (ERC Starting Grant: #757699, B&MGF: INV-001754), OJB (Wellcome Trust: 206471/Z/17/Z), JDM (Wellcome Trust: 210758/Z/18/Z), TWR (Wellcome Trust: 206250/Z/17/Z), KP (B&MGF: INV-003174, European Commission: 101003688), BJQ (NIHR: 16/137/109, NIHR: 16/136/46, B&MGF: OPP1139859), SRM (Wellcome Trust: 210758/Z/18/Z), GFM (B&MGF: NTD Modelling Consortium OPP1184344), EF (MRC: MR/N013638/1), CJVA (ERC: SG 757688), CABP (B&MGF: NTD Modelling Consortium OPP1184344, FCDO/Wellcome Trust: Epidemic Preparedness Coronavirus research programme 221303/Z/20/Z), AE (Nakajima Foundation), JCE (ERC Starting Grant: #757699), QJL (UK MRC: LID DTP MR/N013638/1), RMGJH (ERC Starting Grant: #757699), CD (NIHR: 16/137/109), JYL (B&MGF: INV-003174), KS (Wellcome Trust: 210758/Z/18/Z), DS (BBSRC LIDP: BB/M009513/1), JWR (DTRA: HDTRA1-18-1-0051).

References

1. England, N. H. S. NHS England » Regional teams. https://www.england.nhs.uk/about/regional-area-teams/.
2. Liu, X. *et al.* Safety and immunogenicity of heterologous versus homologous prime-boost schedules with an adenoviral vectored and mRNA COVID-19 vaccine (Com-COV): a single-blind, randomised, non-inferiority trial. *The Lancet* vol. 398, issue 10303, 856-869 (2021) https://doi.org/10.1016/S0140-6736(21)01694-9 doi:10.1016/S0140-6736(21)01694-9.
3. UK Summary. https://coronavirus.data.gov.uk.
4. Willis, K. S. A. Coronavirus (COVID-19) Infection Survey: England. (2021).
5. COVID-19 hub. https://www.ukbiobank.ac.uk/learn-more-about-uk-biobank/covid-19-hub.
6. Real-time Assessment of Community Transmission findings. https://www.imperial.ac.uk/medicine/research-and-impact/groups/react-study/real-time-assessment-of-community-transmission-findings/.
7. Hill, R. Y. A. Coronavirus (COVID-19) Infection Survey, antibody and vaccination data, UK. (2021).
8. Yapp, R. & Willis, Z. Coronavirus (COVID-19) Infection Survey: England. (2022).
9. COVID-19 hub. https://www.ukbiobank.ac.uk/learn-more-about-uk-biobank/covid-19-hub.
10. Statistics. Statistics » COVID-19 Vaccinations. https://www.england.nhs.uk/statistics/statistical-work-areas/covid-19-vaccinations/.
11. Department of Health and Social Care. JCVI advice on the UK vaccine response to the Omicron variant. *GOV.UK* https://www.gov.uk/government/publications/uk-vaccine-response-to-the-omicron-variant-jcvi-advice/jcvi-advice-on-the-uk-vaccine-response-to-the-omicron-variant (2021).

12. JCVI statement on COVID-19 vaccination of children and young people: 22 December 2021. *GOV.UK* https://www.gov.uk/government/publications/jcvi-update-on-advice-for-covid-19-vaccination-of-children-and-young-people/jcvi-statement-on-covid-19-vaccination-of-children-and-young-people-22-december-2021.
13. Barnard, R. C., Davies, N. G., Pearson, C. A. B., Jit, M. & John Edmunds, W. Projected epidemiological consequences of the Omicron SARS-CoV-2 variant in England, December 2021 to April 2022. *medRxiv* 2021.12.15.21267858 (2021).
14. Khoury, D. S. *et al.* Neutralizing antibody levels are highly predictive of immune protection from symptomatic SARS-CoV-2 infection. *Nat. Med.* **27**, 1205–1211 (2021).
15. Scientific Advisory Group for Emergencies. LSHTM: Interim roadmap assessment – prior to steps 3 and 4, 5 May 2021. *GOV.UK* https://www.gov.uk/government/publications/lshtm-interim-roadmap-assessment-prior-to-steps-3-and-4-5-may-2021 (2021).
16. Andrews, N. *et al.* Duration of Protection against Mild and Severe Disease by Covid-19 Vaccines. *N. Engl. J. Med.* **386**, (2022).
17. Davies, N. G. *et al.* Association of tiered restrictions and a second lockdown with COVID-19 deaths and hospital admissions in England: a modelling study. *The Lancet Infectious Diseases* vol. 21 482–492 (2021).
18. Lauer, S. A. *et al.* The Incubation Period of Coronavirus Disease 2019 (COVID-19) From Publicly Reported Confirmed Cases: Estimation and Application. *Ann. Intern. Med.* **172**, 577–582 (2020).
19. Ferretti, L. *et al.* The timing of COVID-19 transmission. *Epidemiology* (2020).
20. Li, Q. *et al.* Early Transmission Dynamics in Wuhan, China, of Novel Coronavirus-Infected Pneumonia. *N. Engl. J. Med.* **382**, 1199–1207 (2020).
21. Nishiura, H., Linton, N. M. & Akhmetzhanov, A. R. Serial interval of novel coronavirus

(COVID-19) infections. *Int. J. Infect. Dis.* **93**, 284–286 (2020).

22. Bi, Q. *et al.* Epidemiology and transmission of COVID-19 in 391 cases and 1286 of their close contacts in Shenzhen, China: a retrospective cohort study. *Lancet Infect. Dis.* **20**, 911–919 (2020).
23. Davies, N. G. *et al.* Age-dependent effects in the transmission and control of COVID-19 epidemics. *Nat. Med.* **26**, 1205–1211 (2020).
24. Davies, N. G. *et al.* Effects of non-pharmaceutical interventions on COVID-19 cases, deaths, and demand for hospital services in the UK: a modelling study. *The Lancet Public Health.* Volume 5, Issue 7, 375-385 (2020) https://doi.org/10.1016/S2468-2667(20)30133-X doi:10.1016/S2468-2667(20)30133-X.
25. Mossong, J. *et al.* Social contacts and mixing patterns relevant to the spread of infectious diseases. *PLoS Med.* **5**, e74 (2008).
26. Population estimates for the UK, England and Wales, Scotland and Northern Ireland - Office for National Statistics. https://www.ons.gov.uk/peoplepopulationandcommunity/populationandmigration/populationestimates/bulletins/annualmidyearpopulationestimates/mid2018.
27. Docherty, A. B. *et al.* Features of 20 133 UK patients in hospital with covid-19 using the ISARIC WHO Clinical Characterisation Protocol: prospective observational cohort study. *BMJ* **369**, (2020).
28. Optimising the COVID-19 vaccination programme for maximum short-term impact. *GOV.UK* https://www.gov.uk/government/publications/prioritising-the-first-covid-19-vaccine-dose-jcvi-statement/optimising-the-covid-19-vaccination-programme-for-maximum-short-term-impact.
