# Supplementary material for: Modelling the medium-term dynamics of SARS-CoV-2 transmission in England in the Omicron era
Source: Nat Commun. 2022 Aug 19;13:4879. doi: 10.1038/s41467-022-32404-y (PMC9389516; doi:10.1038/s41467-022-32404-y)
Supplement: Supplementary file 3 — Reporting Summary [file 41467_2022_32404_MOESM3_ESM.pdf]

nature portfolio

Corresponding author(s): Rosanna C. Barnard

Last updated by author(s): Jul 8, 2022

Reporting Summary

Nature Portfolio wishes to improve the reproducibility of the work that we publish. This form provides structure for consistency and transparency in reporting. For further information on Nature Portfolio policies, see our Editorial Policies and the Editorial Policy Checklist.

Statistics

For all statistical analyses, confirm that the following items are present in the figure legend, table legend, main text, or Methods section.

n/a	Confirmed	
☐	☒	The exact sample size (*n*) for each experimental group/condition, given as a discrete number and unit of measurement
☒	☐	A statement on whether measurements were taken from distinct samples or whether the same sample was measured repeatedly
☒	☐	The statistical test(s) used AND whether they are one- or two-sided<br>*Only common tests should be described solely by name; describe more complex techniques in the Methods section.*
☒	☐	A description of all covariates tested
☒	☐	A description of any assumptions or corrections, such as tests of normality and adjustment for multiple comparisons
☐	☒	A full description of the statistical parameters including central tendency (e.g. means) or other basic estimates (e.g. regression coefficient) AND variation (e.g. standard deviation) or associated estimates of uncertainty (e.g. confidence intervals)
☒	☐	For null hypothesis testing, the test statistic (e.g. *F*, *t*, *r*) with confidence intervals, effect sizes, degrees of freedom and *P* value noted<br>*Give P values as exact values whenever suitable.*
☐	☒	For Bayesian analysis, information on the choice of priors and Markov chain Monte Carlo settings
☒	☐	For hierarchical and complex designs, identification of the appropriate level for tests and full reporting of outcomes
☒	☐	Estimates of effect sizes (e.g. Cohen's *d*, Pearson's *r*), indicating how they were calculated

Our web collection on statistics for biologists contains articles on many of the points above.

Software and code

Policy information about availability of computer code

Data collection	No data collection was performed in this study and therefore no data collection software was used.
Data analysis	All data were analysed and model fits run in R version 4.0.3, with a C++ backend for the model fitting and projections. All of the code required to process data, run model fits, run model projections and produce visualisations has been provided in a public GitHub repository (available at: https://github.com/rosannaclairebarnard/covidm-mtd-Omi). We have included these links in the Data Availability statement in the main manuscript, as well as citing a DOI for the code release within the main manuscript.

For manuscripts utilizing custom algorithms or software that are central to the research but not yet described in published literature, software must be made available to editors and reviewers. We strongly encourage code deposition in a community repository (e.g. GitHub). See the Nature Portfolio guidelines for submitting code & software for further information.

Data

Policy information about availability of data

All manuscripts must include a data availability statement. This statement should provide the following information, where applicable:

- Accession codes, unique identifiers, or web links for publicly available datasets
- A description of any restrictions on data availability
- For clinical datasets or third party data, please ensure that the statement adheres to our policy

Data sources used for these analyses are either publicly available or were provided to the named authors as members of the UK's Scientific Pandemic Influenza Modelling (SPI-M) group, which provides expert advice to the UK's Department of Health and Social Care and wider UK government on scientific matters. Specific permissions have been sought to use SPI-M data for this publication. The SPI-M datasets used for model fitting are unpublished and not publicly available, but are closely aligned with the UK Government's COVID-19 dashboard (https://coronavirus.data.gov.uk/) and other publicly available sources such as the Wellcome Sanger

Institute's COVID-19 genomic surveillance data (https://covid19.sanger.ac.uk/downloads). The following public datasets have been used in this work: school attendance data (https://explore-education-statistics.service.gov.uk/find-statistics/attendance-in-education-and-early-years-settings-during-the-coronavirus-covid-19-outbreak), PCR prevalence data (https://www.ons.gov.uk/peoplepopulationandcommunity/healthandsocialcare/conditionsanddiseases/datasets/coronaviruscovid19infectionsurveydata), seroprevalence data (https://www.ukbiobank.ac.uk/learn-more-about-uk-biobank/covid-19-hub; https://www.imperial.ac.uk/medicine/research-and-impact/groups/react-study/real-time-assessment-of-community-transmission-findings/; https://www.ons.gov.uk/peoplepopulationandcommunity/healthandsocialcare/conditionsanddiseases/datasets/coronaviruscovid19antibodydatafortheuk), mobility data (https://www.google.com/covid19/mobility/), COVID-19 vaccination statistics (https://www.england.nhs.uk/statistics/statistical-work-areas/covid-19-vaccinations/), and data on SARS-CoV-2 lineages in England (https://covid19.sanger.ac.uk/lineages/raw).

Field-specific reporting

Please select the one below that is the best fit for your research. If you are not sure, read the appropriate sections before making your selection.

☒ Life sciences ☐ Behavioural & social sciences ☐ Ecological, evolutionary & environmental sciences

For a reference copy of the document with all sections, see nature.com/documents/nr-reporting-summary-flat.pdf

Life sciences study design

All studies must disclose on these points even when the disclosure is negative.

Sample size	Sample sizes are not directly relevant to this modelling study as we do not collect primary data. Our modelling and analysis relies only on high-powered population-level study estimates (e.g., PCR prevalence and seroprevalence estimates and mobility data) and on individual-level data (e.g., COVID-19 vaccinations, hospital admissions and deaths). These datasets were sufficiently powered to enable the analyses.
Data exclusions	No data were excluded from the study.
Replication	The code used to process data, run model fits, run model projections and produce visualisations has been provided to enable reproducibility. See: https://github.com/rosannaclairebarnard/covidm-mtd-Omi and https://zenodo.org/record/6806637
Randomization	Randomization is not directly relevant to this modelling study as it does not contain study groups. However, model fitting and projection processes rely on random sampling processes.
Blinding	Blinding is not relevant to this modelling study as it does not contain study groups for comparison.

Reporting for specific materials, systems and methods

We require information from authors about some types of materials, experimental systems and methods used in many studies. Here, indicate whether each material, system or method listed is relevant to your study. If you are not sure if a list item applies to your research, read the appropriate section before selecting a response.

Materials & experimental systems

n/a	Involved in the study	
☒	☐	Antibodies
☒	☐	Eukaryotic cell lines
☒	☐	Palaeontology and archaeology
☒	☐	Animals and other organisms
☒	☐	Human research participants
☒	☐	Clinical data
☒	☐	Dual use research of concern

Methods

n/a	Involved in the study	
☒	☐	ChIP-seq
☒	☐	Flow cytometry
☒	☐	MRI-based neuroimaging
