# Supplementary material for: Modelling the medium-term dynamics of SARS-CoV-2 transmission in England in the Omicron era
Source: Nat Commun. 2022 Aug 19;13:4879. doi: 10.1038/s41467-022-32404-y (PMC9389516; doi:10.1038/s41467-022-32404-y)
Supplement: Supplementary file 2 — Peer Review File [file 41467_2022_32404_MOESM2_ESM.pdf]

nature portfolio

Peer Review File

Modelling the medium-term dynamics of SARS-CoV-2 transmission in England in the Omicron era

Open Access This file is licensed under a Creative Commons Attribution 4.0 International License, which permits use, sharing, adaptation, distribution and reproduction in any medium or format, as long as you give appropriate credit to the original author(s) and the source, provide a link to the Creative Commons license, and indicate if changes were made. In the cases where the authors are anonymous, such as is the case for the reports of anonymous peer reviewers, author attribution should be to 'Anonymous Referee' followed by a clear attribution to the source work. The images or other third party material in this file are included in the article's Creative Commons license, unless indicated otherwise in a credit line to the material. If material is not included in the article's Creative Commons license and your intended use is not permitted by statutory regulation or exceeds the permitted use, you will need to obtain permission directly from the copyright holder. To view a copy of this license, visit http://creativecommons.org/licenses/by/4.0/.

Editorial Note: This manuscript has been previously reviewed at another journal that is not operating a transparent peer review scheme. This document only contains reviewer comments and rebuttal letters for versions considered at *Nature Communications*.

REVIEWERS' COMMENTS

Reviewer #1 (Remarks to the Author):

In this revision, the authors have presented a thoroughly updated version of the manuscript accounting for the emergence of the omicron variant. They have expanded the analysis of historical data and provided a thoughtful discussion of how to interpret their findings going into the future. The authors have addressed all of my concerns from the original manuscript, and I have no further concerns with this one.

Reviewer #2 (Remarks to the Author):

The authors have extensively revised their manuscript since their earlier review, adding more detailed description of the modelling framework and key assumptions in response to my earlier queries. I am satisfied that my questions have been comprehensively addressed.They have also updated the work to include the more recently observed Omicron wave and anticipated future epidemiological behaviour. The work's conclusions are supported by the evidence and appropriately caveated in relation to ongoing uncertainties.
